# Dietary Conversion from All-Concentrate to All-Roughage Alters Rumen Bacterial Community Composition and Function in Yak, Cattle-Yak, Tibetan Yellow Cattle and Yellow Cattle

**DOI:** 10.3390/ani14202933

**Published:** 2024-10-11

**Authors:** Yili Liu, Yu Wang, Yongli Wen, Liangliang Ma, Daojie Riqing, Mingfeng Jiang

**Affiliations:** 1 Key Laboratory of Qinghai-Tibetan Plateau Animal Genetic Resource Reservation, College of Animal Science and Veterinary Medicine, Southwest Minzu University, Chengdu 610041, China; yililiunum@163.com (Y.L.); nottwya@gmail.com (Y.W.); wansit99@163.com (Y.W.); 19822966564@163.com (D.R.); 2 College of Grassland Resources, Southwest Minzu University, Chengdu 610041, China; maliangliang2020@163.com

**Keywords:** yak, cattle-yak, all-concentrate, all-roughage, rumen, metagenomics

## Abstract

**Simple Summary:**

Dietary composition is the main factor regulating the rumen microflora structure and fermentation mode. The change of the concentrate to roughage ratio of diets can cause the change of rumen microflora and affect the feed utilization and growth performance of ruminants. This study investigated the effects of the extreme transition from all-concentrate to all-roughage diets on the patterns and functions of the rumen microbiome in high-altitude yaks, cattle-yak, Tibetan yellow cattle and low-altitude yellow cattle by metagenomic sequencing. After diet conversion, the rumen microbial richness and diversity of the four herds increased, and the biggest difference between concentrate and roughage diets was yak and cattle-yak, followed by Tibetan yellow cattle and yellow cattle. The transformation in diets can change the metabolic pathways of rumen microorganisms in four herds and finally affect the fermentation mode of rumen. Compared with Tibetan yellow cattle and yellow cattle, yaks and cattle-yaks have better adaptability to roughage, and its utilization rate can be fully improved to reduce methane emission, and they are more suitable for intensive feeding on the plateau.

**Abstract:**

The experiment was to compare the effects of switching all-concentrate to all-roughage diets on rumen microflora and functional metabolism of yak, cattle-yak, Tibetan yellow cattle and yellow cattle living in different altitudes. A total of 24 yaks, cattle-yaks, Tibetan yellow cattle and yellow cattle with a similar weight and good body condition aged 3.5 years were selected and divided into four groups according to species. They were fed a concentrate diet with 40% soybean meal and 60% corn meal for the first month (C group) and a roughage diet with dry corn stalks (100%) for the second month (R group); the formal experimental period was 60 d. These results showed that the conversion had a significant effect on the rumen microflora structure of the four herds, and the biggest difference between concentrate and roughage diets was yak and cattle-yak, followed by Tibetan yellow cattle and yellow cattle. At the phylum level, Bacteroidetes and Firmicutes still predominate in all groups. Compared with the C groups, the relative abundance of Lentisphaerae and Kiritimatiellaeota increased in all R groups, and Lentisphaerae was significantly increased in yak and cattle-yak (*p* < 0.05). At the genus and species levels, *Prevotella* had the highest abundance, and the relative abundances of *Prevotella, Ruminococcus, Sarcina* and *Ruminobacter* in R groups were lower, while the abundances of other differential genera, including *Methanobrevibacter, Fibrobacter*, *Treponema*, *Eubacterium*, *Butyrivibrio*, *Succinivibrio* and *Succinimonas*, were all higher. Roughage diets increased the number of unique genes and functional genes encoding different CAZymes in rumen microorganisms in all four herds. In the functional contribution analysis, with the exception of ABC transporters and methane metabolism, *Prevotella* was the main contributor to almost all of these functions. In methane metabolism, *Methanobrevibacter* had the highest relative abundance, followed by *Prevotella*, Clostridia and Bacteroidales in all groups. Compared with Tibetan yellow cattle and yellow cattle, yaks and cattle-yaks have better adaptability to roughage, and its utilization rate can be fully improved to reduce methane emission. The study indicates that when four herds are converted to high roughage at the later stage of feeding, the growth and reproduction of rumen microorganisms are affected, and the abundance and diversity of rumen microorganisms are increased to varying degrees. The transformation of concentrate to roughage diet can change the metabolic pathways of rumen microorganisms in yaks and finally affect the fermentation mode of rumen. The above results provide a theoretical basis for the research and development of fattening feeds for yaks, cattle-yaks, Tibetan yellow cattle and yellow cattle and the intensive feeding of livestock on the plateau.

## 1. Introduction

Known as “the roof of the world”, the world’s “Third Pole” and “Water Tower of Asia”, the Qinghai–Tibetan Plateau (QTP) is the highest and largest plateau in the world, with mean altitude above 4500 m asl and with an area over 2.5 million km^2^ [1,2]. The grassland ecosystems of more than 3500 m, which covers about 60 percent of the QTP, provide an important habitat for many ruminant species, which utilize pasture for grazing throughout the year [1,3,4,5]. In harsh environments, these ruminants such as yaks, cattle-yaks and Tibetan yellow cattle have evolved both physiologically and morphologically to withstand the harsh environmental conditions, especially high altitude, cold, strong UV radiation, low oxygen partial pressure, high food scarcity, etc. [6,7]. Among domesticated ruminants, yak (*Bos grunniens*) is a unique and significant species in the QTP region, which can adapt well to the low-temperature and low-oxygen environment (3000–5500 m), and was domesticated by early nomadic people more than 7300 years ago [8,9]. Cattle-yaks, the F1 hybrid offsprings of yak and cattle (*Bos taurus*), inhabit the QTP region at an altitude of above 3000 m and obtain nutrients mainly from grazing on natural grasslands [10,11]. Cattle-yak, an important domestic animal in the QTP region, is more resilient to harsh environments and can provide more meat, milk, wool, fuel and other products per weight than the yak [12,13]. Indigenous Tibetan yellow cattle originated on the QTP region are mainly raised outdoors on natural pastures [14]. Although in nature, three plateau ruminants graze on natural pastures all year round with little to no replenishment, they are severely challenged by the scarcity of grassland resources in the QTP areas during cold seasons [12,15,16]. Therefore, they must have evolved the underlying mechanisms to adapt to the harsh environment. The rumen of ruminants contain microorganisms with lignocellulosic degrading capabilities, enabling them to adapt to the plateau environment [17,18]. Unlike low-altitude ruminants, the scarcity of natural pasture at high altitude allows ruminants to extract more nutrients from forage by increasing the residence time of rumen habitat microorganisms and the ability to produce a range of enzymes to utilize fibrous feed [19,20,21].

Rumen is an important digestive organ for ruminants, such as yak, cattle-yak and sheep [22]. Rumen bacteria play a key role in the dietary energy digestion of plateau ruminants and adaptation to the plateau environment. Ruminants have a diverse microbiota in the rumen including bacteria, fungi, archaea, protozoa and viruses [23,24], which can degrade the plant cell walls and fibrous substances into absorbable compounds such as proteins and volatile fatty acids (VFAs) [25,26]. Yaks, cattle-yaks and Tibetan yellow cattle have adapted to this harsh high-altitude environment, and adaptive evolution has affected genes related to energy metabolism, thereby helping these ruminants survive at high altitudes [24,27]. Compared with ruminants at low altitudes, ruminants on plateau not only evolve their host genomes but also host microbial genomes [17,18]. Such studies have shown that the convergent evolution of rumen microbiomes for energy harvesting persisted in yaks and T-sheep, two typical high-altitude ruminants, with greatly lower levels of methane and significantly higher VFA production than low-altitude cattle and ordinary sheep [17]. The bacterial diversity of Holstein cattle, yellow cattle and three yak herds in the QTP area was significantly different, indicating that host genetics and geography affected the stomach compartment microbiota [28]. These results not only enhance our understanding of the role of microbiota in the nutrition and metabolism of plateau ruminants, thereby providing new insights into nutrition management but also improve our understanding of the adaptation of plateau ruminants. Exploring the microbiota between cattle and yak in different geographies could deepen our understanding of the role of microbiota in metabolism and adaptation.

The rumen is a complex microbial environment containing a variety of microorganisms that help the host to digest and utilize feed energy [29,30,31]. The growth and survival of ruminants are largely dependent on metabolites produced by microorganisms in the rumen [32,33]. Therefore, a better understanding of the rumen microbiota fed with different diet types is critical for exploiting the microbiota to improve feed efficiency [34,35]. Feed efficiency is an important management method to improve the economy of meat and milk production [36,37]. A study conducted on yaks reported the difference in rumen microbiota and certain metabolites when fed different feed types divided into roughage and concentrate groups [38]. When fed with varying ratios of roughage to concentrate mix, a high-roughage diet can selectively enrich microbial communities capable of metabolizing aromatic compounds in the rumen of domestic buffalo [39]. However, little information is available regarding the impact of different feeding types on dynamic changes in the rumen fermentation and microbiota in the rumen of different cattle breeds at different altitudes at the same time [18,19,40], especially among high-altitude domestic animals such as yak, Tibetan yellow cattle and cattle-yak. These plateau livestock are basically raised by the traditional way of full grazing without replenishment, due to the extensive husbandry and management of herdsmen, resulting in a long husbandry cycle, low stocking rate, slow turnover and low breeding efficiency, which greatly restricts the economic development of pastoral areas [41]. In order to improve this situation, in recent years, breeding methods such as house feeding and semi-house feeding have been promoted, such as the standardized yak breeding “4218” model [42], which has greatly promoted the development of yak industry. Therefore, it is important to clarify the effects of different diet types on the changes of rumen microbial types and functions of high-altitude livestock, which is conducive to the development of standardized scale farming. This study aims to explore the effects of the conversion of all-concentrate to all-roughage on the rumen microbial fermentation, bacterial composition and function of high-altitude yak, Tibetan yellow cattle, cattle-yak and low-altitude yellow cattle and provide theoretical support for the subsequent large-scale intensive breeding of yak, Tibetan yellow cattle and cattle-yak.

In this study, the Illumina Hi-Seq platform was used to conduct metagomic sequencing analysis, and four cattle breeds at different altitudes were fed in the same farm for 2 months to adapt to the environment and then fed with all-concentrate diets (40% soybean meal and 60% corn meal) and all-roughage diets (100% corn stalks) for 1 month, respectively. To observe the effects of the extreme dietary conversion from all-concentrate to all-roughage on the structure and function of rumen microflora in high-altitude yak, Tibetan yellow cattle, cattle-yak and low-altitude yellow cattle were observed. This study will not only provide data support for the regulation of concentrate and roughage diet conversion in yaks, cow-yaks, Tibetan cattle and cattle rumen flora but also lay a theoretical foundation for the large-scale intensive feeding of high-altitude livestock.

## 2. Materials and Methods

### 2.1. Ethics Statement

All experimental procedures involving animals were reviewed and approved by the Institutional Animal Care and Use Committee at Southwest Minzu University (Chengdu, Sichuan, China), and all studies were in line with the requirements of the directory of the Ethical Treatment of Experimental Animals of China.

### 2.2. Experimental Design and Sample Collection

**Figure 1:**
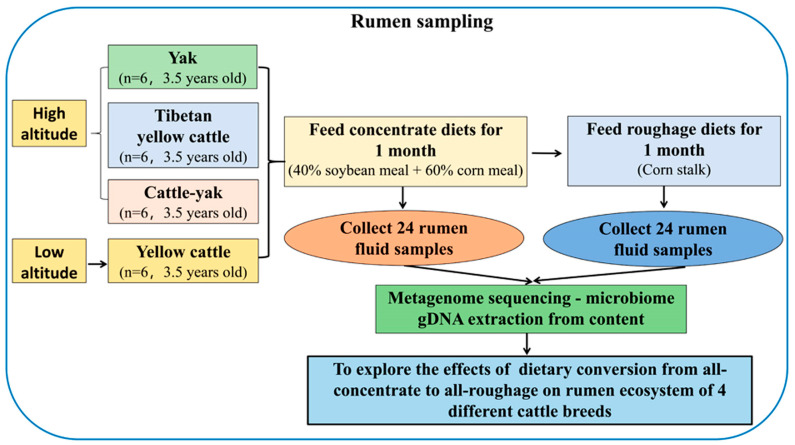
Schematic diagram of experimental design.

There were 24 experimental cattle, including yak, cattle-yak, Tibetan yellow cattle and yellow cattle. All the experimental animals were purchased from different altitudes, and each breed of cattle came from the same place and were randomly selected from a large group. Animals of similar age, health and weight were selected, with 6 animals in each group. The selection criteria were as follows: age 3.5 years, average weight 209.8 ± 41.98 kg, male and female half. In order to reduce the differences between groups and individuals in the follow-up experiment, the purchased cattle were fed intensively at Qingning Yak farm in Jinchuan County, Aba Tibetan and Qiang Autonomous Prefecture, Sichuan Province, for 2 months before the formal experiment began. No antibiotics were used before the experiment. Each group was fed either concentrate and roughage diets successively. The 100% concentrate diet is soybean meal (40%) and corn meal (60%), and the 100% roughage is corn stalks. The experimental design is shown in Figure 1.

In each of the four groups, animals received the concentrate diets for 30 days followed by the roughage diets for 30 days. These animals were maintained on each diet for the 30-day time period in each case to allow for microbial adhesion and adaptation to the diet in the rumen. After each feeding period, rumen samples were collected using a negative pressure collector. Each of the four experimental groups were further divided into two subgroups that were fed with concentrate mix to the roughage. There were 8 groups as in Table 1. There were, therefore, 48 samples in total in this study. Samples were then separated into solid and liquid fractions by filtering through 4 layers of sterile gauze. Each sample was immediately placed in dry ice and transported to the laboratory. Each rumen fluid sample was filtered through four layers of sterile cheesecloth, and 100 mL of filtered rumen fluid was stored in liquid nitrogen until further analysis.

### 2.3. Determination of Rumen Carbohydrate Enzyme Activity and Fermentation Parameters

The 48 samples collected were sent to Logos (Jinan) Biomedical Co., Ltd. (Jinan, China), for carbohydrate enzyme activity determination. Volatile fatty acids in rumen fluid were detected by liquid chromatography. Take 1.0 mL sample, add 3.0 mL distilled water, mix well, centrifuge at 8000 r/min for 10 min, and put the supernatant through 0.22 μm microporous filter membrane before feeding. Mass spectrometer parameters: (1) chromatographic column: Philomene C18 column 250 mm × 4.6 mm, 5 μm; (2) flow rate: 1.0 m L/min; (3) wavelength: 214 nm; (4) column temperature: 25 °C; (5) mobile phase: A-(0.2% phosphoric acid solution), B-methanol.

### 2.4. DNA Extraction and High-Throughput Sequencing

The 48 samples collected were sent to DeepBiome Co., Ltd. (Shanghai, China), for metagenomic sequencing and functional analysis. The microbial genomic DNA was extracted from each sample using a PowerFecalTM Fecal DNA Kit (MOBIO, Carlsbad, CA, USA) according to the manufacturer’s instructions. The extracted metagenomic DNA samples were measured on a nanodrop spectrophotometer to assess quantity. The quantity and quality of these libraries were examined using an Agilent 2100 Bioanalyzer (Agilent Technologies, Palo Alto, CA, USA), and a 350 bp paired-end sequencing library was constructed via NGS Fast DNA Library Prep Set for Illumina (Mei5 Biotechnology, Co., Ltd., Beijing, China). The qualified libraries were sequenced using the Illumina HiSeq sequencing platform.

### 2.5. Data Quality Control

The raw reads were extracted from the sequencing results, and the quality of the raw reads was assessed using FastQC (version 0.11.9) with default parameters [43]. Adaptor sequences in the raw reads were trimmed using Trimmomatic (Version 0.38) [44]. Moreover, the bases at the begin and end of each read with quality below 3 were cut. The Trimmomatic slides from the 5′ end in windows, and the window size was 4 bases; when the average quality in the window is lower than the setting threshold of 15, the read is cut. Only the reads with length >36 bp were kept. The BMTagger (version 3.102) was used to remove the host genome sequence, which used GCF_000298355.1_BosGru_v2.0 (for yak samples), GCA_000003205.6_Btau_5.0.1 (for cattle and cattle-yak samples) and Enterobacteria phage phiX174 reference genome to build the BMTagger index to complete the filtering of the host sequence.

### 2.6. Taxonomy Profiling and Assembly

Braken2 (version 2.0.8b) [45], Kaiju (version 1.6.3) [46] and Kraken were involved in the taxonomic classification of the metagenome short reads. GTDB (version 04-RS89) [47] database collects 24,706 bacterial and archaeal representative genomes, and additional redundant genomes collected from other databases were added to increase classification rate [48]. A total of 53,346 genomes were retrieved from GenBank, Refseq and GTDB databases to build Kraken2 database. The Braken2 species abundance was assessed using the read length of 100, 150 and 200 bp deposit in Kracken (version 2.5.0) [49] database based on the standard procedure of Kraken2 database. The latest Kaiju index database (25 June 2019) was downloaded from Kaiju website (http://kaiju.binf.ku.dk/server, accessed on November 2018) and used for Kaiju species abundance estimation. The relative abundance tables from Kaiju, Kraken or Bracken were used for Principal Component Analysis (PCA), Principal Co-ordinates Analysis (PCoA) heatmap, Non-Metric Multi-Dimensional Scaling (NMDS) and bar plot visualization, respectively. The raw-read count tables at different taxonomic levels (phylum, order, class, family, genus, species) estimated by Kaiju, Kraken or Bracken classifier were used for differential abundance analyses with DEseq2 [50].

Meta-seq pipeline uses metaSPAdes (version 3.13.2) [51] and MEGAHIT (version 1.2.9) [52] for short-read assembly. The assembly parameters and error correction step used with metaSPAdes were set as “—meta –threads 40 –k -k 21, 33, 55”. De bruijn- graph-based assembler MEGAHIT was employed to assemble short reads with the parameters of -cpu-only -presets meta-sensitive -m 200000000000. For the unaligned reads or contigs < 200 bp in all samples, they were collected for further assembly with the same assembly parameter. All contigs > 200 bp in length were used to construct a reference set for further bowtie alignment.

### 2.7. Gene Prediction and Functional Profiling

MetaProdigal (version: 2.6.3) was used for the gene prediction of assembled metagenomic contigs [53]. The abundance of each ORF was quantified with Salmon (version 1.1.0) [54,55]. The functional analysis of ORFs was described by KEGG [56,57] and COG. EggNOG-mapper (version 0.12.7) was employed for COG term assignment [58]. KEGG ortholog assignment for ORFs was based on profile HMM using HMMSCAN (HMMER version 3.1b2) [59] with parameter of -e 1e-5.

Using the melt subcommand from tsv-utils (version 0.0.1, https://github.com/jameslz/biostack-suits, accessed on October 2018), the ORFs’ level abundance values (counts and TPM) were binned into corresponding functional catalogs based on KEGG, COG and AMR ontology structure. KEGG Ortholog (KO), module, pathway and catalog (only includes metabolism part) were used as functional levels for KEGG. COG abundance was binned to catalog the classification system. The abundance profiling with TPM values were used for PCA, NMDS, PCoA analyses with heatmap and bar plot visualization. For taxonomic and functional raw count profiling, R package DESseq2 was employed to detect the differential abundance ontology or taxonomy catalog [50].

### 2.8. PCR Validation of Rumen Cellulase Gene

The xylanase GH10 genes obtained by rumen metagenomic sequencing were amplified by PCR, and 2 genes were amplified from each sample. If some gene amplification was not successful, the primers would be redesigned. Primers of 112 genes were designed, and the sequence of primers is shown in Table S1. The above genes were verified by PCR, and the products were detected by 1% agarose gel electrophoresis. The PCR amplification system was 25 µL, T3 Super PCR Mix: 22 µL, upper and downstream primers: 1 µL (0.4 µM) each and template DNA: 1 µL (20 ng/µL). The operation conditions of PCR amplification were as follows: 98 °C, denaturation for 3 min; run at 98 °C for 10 s, 60 °C for 10 s, 72 °C for 45 s, run 30 cycles; 72 °C, 2 min. The final retention temperature was set to 10 °C. Tsingke T3 polymerase was purchased from Tsingke Biotech Co., Ltd. (Chengdu, China).

### 2.9. Data Analysis

All experiments results had at least three replicates and were expressed as mean ± standard deviation (S.D.). ANOVA (SPSS 18.0) was used for statistical evaluation, *p* value < 0.05 was significant and *p* value < 0.01 was extremely significant.

## 3. Results

### 3.1. The Effects of Treatments on Carbohydrate Enzyme Activity

The rumen CAZy activity was analyzed under the conversion of concentrate into roughage diets, and a total of four kinds of CAZy activity were detected. The results are shown in Table 2. The cellobiase activity in the YK group was significantly decreased (*p* < 0.05); the activity of carboxymethyl cellulase in the CY group was significantly decreased (*p* < 0.01) and the activities of cellobiase and microcrystalline cellulase were markedly reduced (*p* < 0.05). The cellobiase activity and microcrystalline cellulase activity in the LC group were greatly decreased (*p* < 0.05). The cellobiase activity and microcrystalline cellulase activity in the HC group were hugely decreased (*p* < 0.01), and the xylanase activity was significantly decreased (*p* < 0.05).

### 3.2. The Effects of Treatments on Rumen Fermentation Parameters

Rumen fermentation parameters were measured under the condition of the conversion of concentrate into roughage diets, and the results were shown in Table 3. In the YK and CY groups, the concentrations of acetate, propionate, butyrate, isovalerate and NH3-N were increased, and butyrate was significantly increased (*p* < 0.05), while isobutyrate and valerate were decreased, but not significantly (*p* > 0.05). In the LC group, the concentrations of acetate, propionate, isobutyrate, butyrate and valerate were all reduced, but not significantly (*p* > 0.05), while the concentrations of isovalerate and NH3-N were all increased, and the NH3-N concentration was markedly increased (*p* < 0.05). In the HC group, acetate, propionate, isobutyrate, isovalerate, valerate and NH3-N concentrations decreased, among which isobutyrate and isovalerate were greatly decreased (*p* < 0.05), while butyrate concentration was increased, but not significantly (*p* > 0.05).

### 3.3. Profiling of the Rumen Metagenome

Metagenome sequencing analysis was performed on the 48 rumen fluid samples from yak, cattle-yak, Tibetan yellow cattle and cattle, and 323 Gb raw data were obtained. After filtering the host genome data, a total of 5.96 × 10^11^ clean bases were obtained for all samples, as shown in Table S2. A total of 14.9 GB contigs with lengths from 36 to 150 bp were obtained.

**Figure 2:**
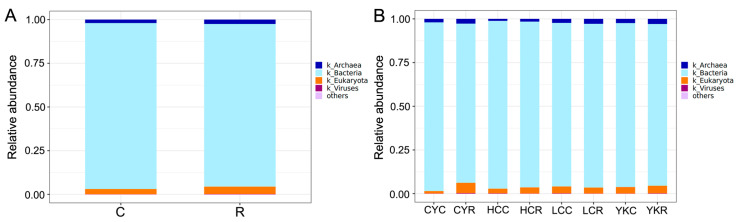
Comparison of rumen microbiota at the domain level. (**A**) Group by roughage and concentrate diets. (**B**) Group by species.

A total of five domains, five kingdoms, 198 phyla, 241 classes, 557 orders, 968 families, and 2311 genera were obtained. At the domain level, bacteria (93.55~96.54% in four C groups, and 91.14~94.97% in four R groups) and Eukaryota (1.39~3.96% in four C groups, and 3.32~5.92% in four R groups) were the most highly abundant taxa, followed by Archaea, viruses and unclassiified taxa (Figure 2 and Table S3).

### 3.4. Comparison of Rumen Microbiome after Feeding Concentrate into Roughage

**Figure 3:**
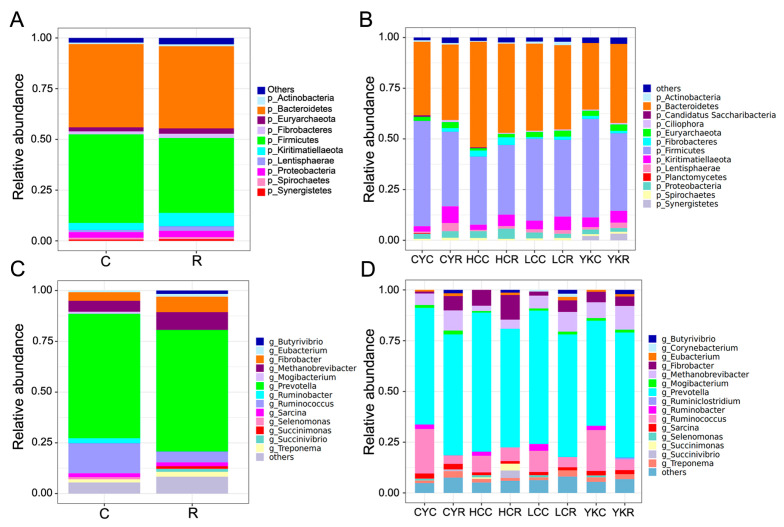
Comparison of the results of metagenomic sequencing of major species at the phylum and genus level. (**A**) Group by concentrate and roughage diets at the phylum level. (**B**) Group by species at the phylum level. (**C**) Group by concentrate and roughage diets at the genus level. (**D**) Group by species at the genus level.

The dominant bacterial phyla in C and R groups included Bacteroidetes (40.99%, 40.53%), Firmicutes (43.72%, 36.85%), followed by Kiritimatiellaeota (3.48%, 6.53%), Proteobacteria (2.86%, 3.06%), Euryarchaeota (2.02%, 2.64%), Lentisphaerae (0.97%, 2.35%) and Fibrobacteres (1.44%, 2.10%) (Figure 3A and Table S4). Compared with the C group, the abundance of Bacteroidetes and Firmicutes decreased in the R group, while the abundance of other dominant bacterial phyla increased. In yaks, the dominant bacterial phyla were Firmicutes (48.57%, 38.31%) and Bacteroidetes (32.83%, 38.98%), followed by Kiritimatiellaeota (4.73%, 5.73%), Euryarchaeota (2.54%, 3.15%), Synergistetes (2.04%, 3.20%), Proteobacteria (2.33%, 1.95%) and Lentisphaerae (1.12%, 2.66%). Compared with the C group, the relative abundance of Lentisphaerae in the R group was significantly increased in yaks (*p* < 0.05). In cattle-yak, the dominant bacterial phyla were Firmicutes (51.98%, 36.73%), Bacteroidetes (36.26%, 37.21%), Proteobacteria (2.60%, 3.26%), Kiritimatiellaeota (2.57%, 8.32%) and Euryarchaeota (2.04%, 2.90%). Among these, the relative abundance of Kiritimatiellaeota, Fibrobacteres and Lentisphaerae in the R group was significantly increased (*p* < 0.01). In Tibetan yellow cattle, the dominant bacterial phyla in C and R groups were Bacteroidetes (52.01%, 43.94%), Firmicutes (33.67%, 34.46%), Kiritimatiellaeota (2.55%, 5.55%), Proteobacteria (3.40%, 5.06%) and Fibrobacteres (2.94%, 3.80%), and the relative abundance of Bacteroidetes in the R group was significantly decreased (*p* < 0.01). In yellow cattle, the dominant bacterial phyla in the C and R groups were Bacteroidetes (42.91%, 41.54%), Firmicutes (40.47%, 37.86%), Kiritimatiellaeota (4.19%, 6.68%), Proteobacteria (3.00%, 2.06%) and Euryarchaea (2.48%, 2.95%), and the relative abundance of Fibrobacteres in the R group was significantly increased (*p* < 0.05) (Figure 3B and Table S3).

The dominant bacterial genera in the C and R groups were *Prevotella* (61.23%, 59.92%), *Ruminococcus* (14.89%, 5.39%), *Methanobrevibacter* (5.38%, 8.71%), *Fibrobacter* (4.24%, 7.61%), *Sarcina* (1.88%, 1.82%), *Treponema* (1.67%, 2.43%) and *Eubacterium* (0.81%, 1.35%) (Figure 3C and Table S4). Compared with the C group, the relative abundances of *Prevotella, Ruminococcus, Sarcina* and *Ruminobacter* in the R group were lower, while the abundances of other differential genera, including *Methanobrevibacter*, *Fibrobacter*, *Treponema*, *Eubacterium*, *Butyrivibrio*, *Succinivibrio* and *Succinimonas*, were all higher. Compared with the C group, under roughage feeding conditions, the relative abundance of *Ruminococcus* in yak significantly decreased (*p* < 0.05), while the *Butyrivibrio* markedly increased (*p* < 0.05); the relative abundance of *Fibrobacter* in cattle-yak greatly increased (*p* < 0.01), but *Ruminobacter* and *Ruminococcus* significantly decreased (*p* < 0.05); the relative abundance of *Prevotella* in Tibetan yellow cattle dramatically reduced (*p* < 0.01), and *Ruminococcus*, *Ruminobacter*, *Treponema*, *Sarcina* and *Mogibacterium* also decreased, and *Fibrobacter*, *Methanobrevibacter*, *Eubacterium* and *Butyrivibrio* increased, but none of them were significant; the relative abundance of *Ruminococcus* and *Selenomonas* in yellow cattle dropped significantly (*p* < 0.05), while *Fibrobacter* and *Eubacterium* markedly boosted (*p* < 0.05) (Figure 3C and Table S5). It was also found that the relative abundances of *Methanobrevibacter* in the four roughage groups were increased, but not significantly, compared with the concentrate groups.

### 3.5. Functional Gene Annotation Analysis

#### 3.5.1. Gene Number Differences between Groups

**Figure 4:**
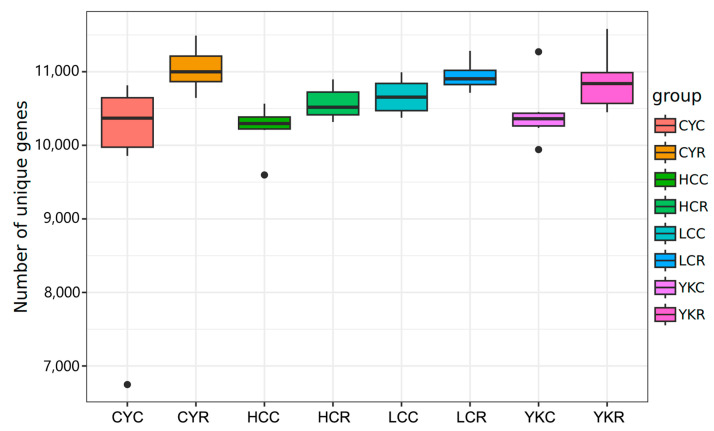
Box plot of gene number differences between groups.

**Figure 5:**
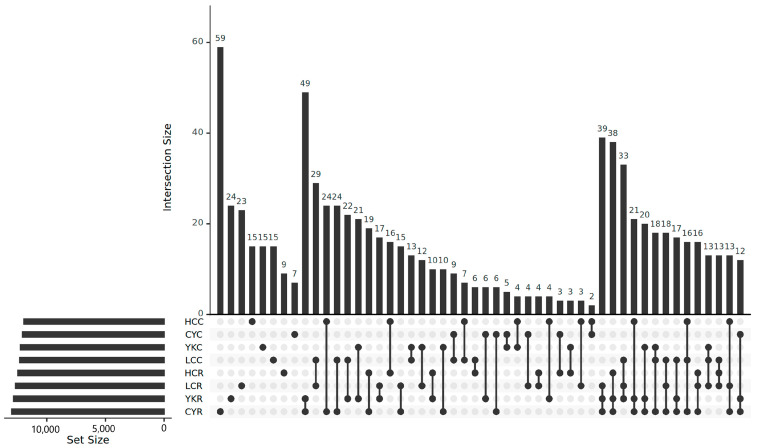
Upset plot graph shows the number of intersection genes.

In cattle-yak, Tibetan yellow cattle, yellow cattle and yak, the number of genes in group C is generally lower than that in group R, and the biggest difference between the concentrate and roughage groups is yak and cattle-yak, while the smaller difference is Tibetan yellow cattle and yellow cattle (Figure 4). The number of genes in the C and R groups of the four species were analyzed by the Venn diagram, respectively, and the results showed that the number of unique genes in cattle-yak was the largest difference (245 vs. 1149), while that in yellow cattle was the smallest (404 vs. 754) (Table 4). In addition, unique microbial genes were found in each group, including 59 genes in CYR, 24 genes in YKR, 23 genes in LCR, 23 genes in LCR, 15 genes in HCC, YKC and LCC, 9 genes in HCR and 7 genes in CYC (Figure 5).

#### 3.5.2. CAZyme Functional Annotation

**Figure 6:**
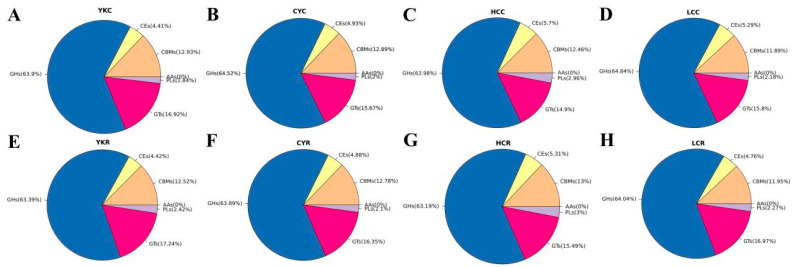
Different CAZy family distribution maps. (**A**–**D**) YKC, CYC, HCC and LCC. (**E**–**H**) YKR, CYR, HCR and LCR.

**Figure 7:**
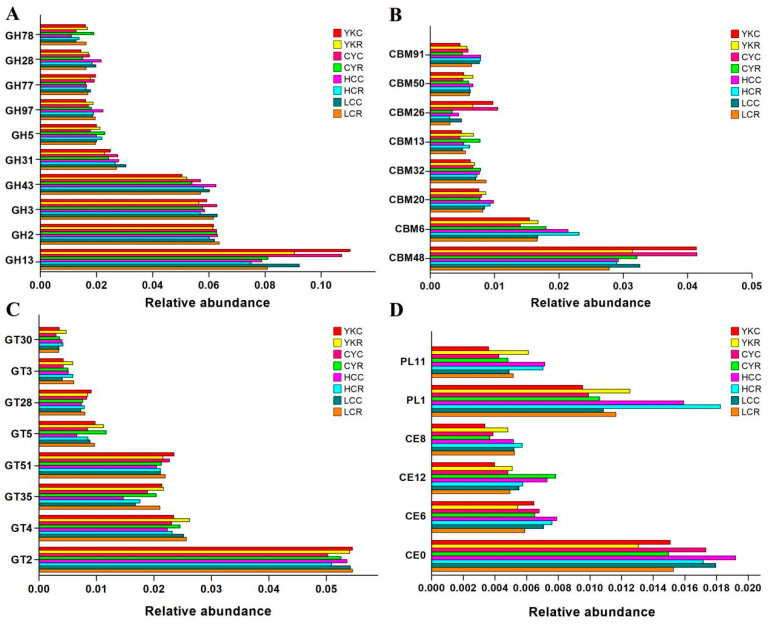
Relative abundance of rumen microbial functional genes encoding CAZymes (TOP 50). (**A**) GHs (TOP10). (**B**) CBMs. (**C**) GTs. (**D**) PLs and CEs.

The rumen metagenomic distribution of the CAZy family in yaks, cattle-yak, Tibetan yellow cattle and yellow cattle is shown in Figure 6. In the eight groups, the relative abundances of six families of CAZy were GHs, GTs, CBMs, CEs, PLs and AAs from high to low. When the diet changed from concentrate to roughage, the relative abundance of the GHs family decreased, and the decrease was the smallest in yak and the largest in yellow cattle. The relative abundance of the GHs family decreased when the diet changed from concentrate to roughage, the decrease was the smallest in yak and the largest in yellow cattle. The relative abundance of the GTs family increased, with the largest increase among yellow cattle, followed by cattle-yak, Tibetan yellow cattle and yak. The relative abundance of the CBMs family in cattle-yak and yak reduced, while the relative abundance of cattle-yak and Tibetan yellow cattle increased. The relative abundance of the GEs family raised in yak but decreased in cattle-yak, Tibetan yellow cattle and yellow cattle, with the most significant decline in yellow cattle (Figure 6). Compared with the C groups, the number of functional genes encoding different CAZymes in the R groups increased (Tables S6 and S7). The TOP 50 CAZy family members in relative abundance include 28 GHs, 8 CBMs, 8 GTs, 4 CEs and 2 PLs. The highest relative abundance is GH13 in the GHs family, CBM48 in the CBMs family, CE0 in the CEs family, GT2 in the GTs family, and PL1 in the PLs family. Compared with the C groups, the relative abundances of CBM48, CBM26, GH13, GH3, GH31, GH77, CE0 and CE6 in the four R groups all declined, the relative abundances of GT4, GT5, GT3 and PL1 increased, while the relative abundance of GT2 decreased in yak and Tibetan yellow cattle and increased in cattle-yak and yellow cattle (Figure 7).

#### 3.5.3. KEGG Functional Annotation

**Figure 8:**
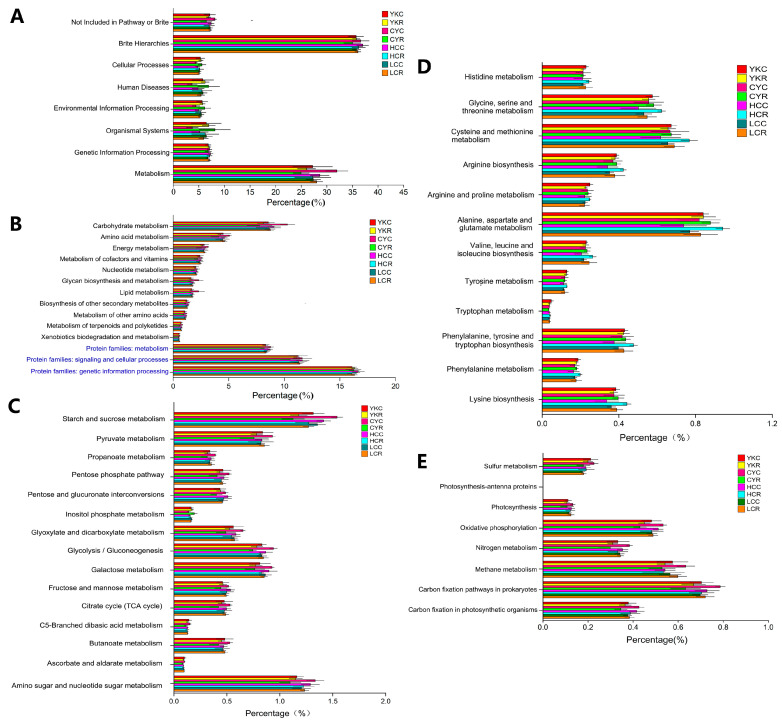
Composition of KEGG gene families among four R (YKR, CYR, HCR and LCR) and C groups (YKC, CYC, HCC and LCC). (**A**) KEGG pathways at level 1. (**B**) KEGG pathways at level 2. (**C**) Carbohydrate metabolism pathways. (**D**) Amino acid metabolism pathways. (**E**) Lipid metabolism pathways.

At level 1, KEGG pathways related to Brite hierarchies (34.97%~36.98%) and metabolism (24.96%~31.95%) were more abundant in eight groups (Figure 8A). Compared with the C groups, the percentage of KEGG pathways related to metabolism in YKR, CYR and HCR was reduced but increased in LCR (Figure 8A). The percentage of KEGG pathways related to metabolism in cattle-yak was the highest among four C groups, but the lowest among the four R groups (Figure 8A). At the KEGG level 2, a total of 41 gene families were identified in eight groups. Of these 41 gene families, the majority belonged to protein families: genetic information processing (16.07–16.64%; Brite hierarchies), protein families: signaling and cellular processes (10.52–11.62%; Brite hierarchies), carbohydrate metabolism (7.77–10.28%; metabolism), protein families: metabolism (8.19–8.76%; Brite hierarchies) and amino acid metabolism (4.08–5.07%; metabolism) in eight groups, followed by energy metabolism, the metabolism of cofactors and vitamins, nucleotide metabolism, etc. (Figure 8B). Compared with the C groups, the relative abundances of carbohydrate metabolism, amino acid metabolism, energy metabolism, the metabolism of cofactors and vitamins and nucleotide metabolism in YKR, CYR and HCR decreased, while those in LCR increased. The relative abundances of carbohydrate metabolism, amino acid metabolism and energy metabolism in CYC were greater than those in YKC, HCC and LCC. The relative abundances of carbohydrate, amino acid metabolism and energy metabolism in LCR were higher than that in YKR, CYR and HCR (Figure 8B).

The KEGG pathways of carbohydrate metabolism, energy metabolism and amino acid metabolism in each group were further analyzed at level 3 (Figure 8C–E). Compared with the C groups, the relative abundances of citrate cycle in all R groups decreased, but the difference was very significant in yaks (*p* < 0.01); the relative abundances of butanoate metabolism in YKR, CYR and HCR reduced, while that in LCR enhanced, but the difference was not significant (Figure 8C). With respect to glycolysis/gluconeogenesis, compared with the C groups, the relative abundances in YKR and CYR were markedly increased (*p* < 0.05), that in HCR was increased, and that in LCR was decreased, but the differences were not significant. The relative abundance of propanoate metabolism decreased in all R groups, but the difference was significant only in CYR (*p* < 0.05) (Figure 8C). Compared with the C groups, the relative abundance of methane metabolism increased in CYR, YKR and HCR and reduced in LCR, but the differences were not notable (Figure 8E).

#### 3.5.4. Functional Contribution Analysis

**Figure 9:**
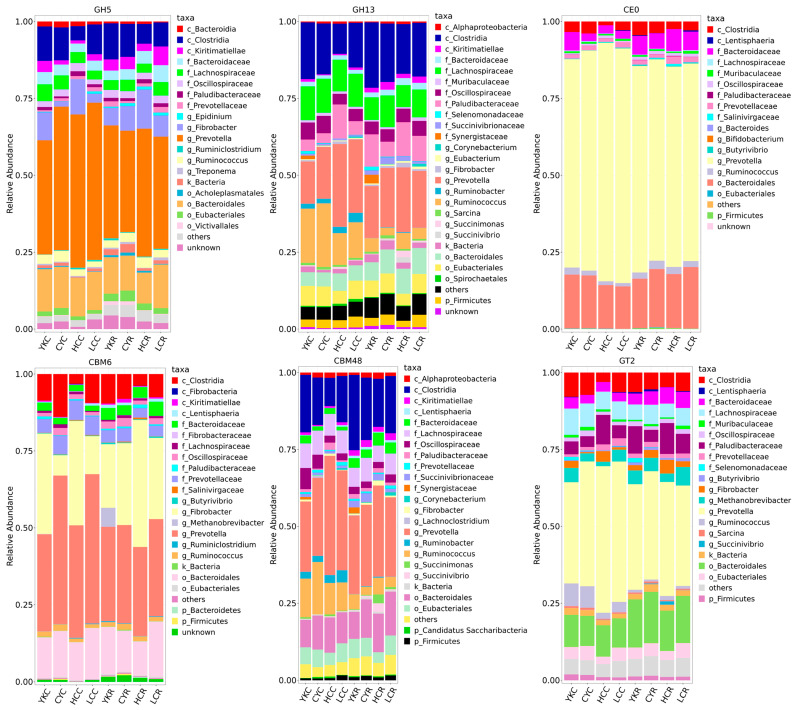
Relative contribution of different taxa to identified rumen-enriched functional attributes of CAZymes encoded genes (GH5, GH13, CE0, CBM6, CBM48 and GT2) at the family level in different samples. GH: glycoside hydrolases; GT: glycosyltransferase; CE: carbohydrate esterases; CBM: carbohydrate-binding modules.

**Figure 10:**
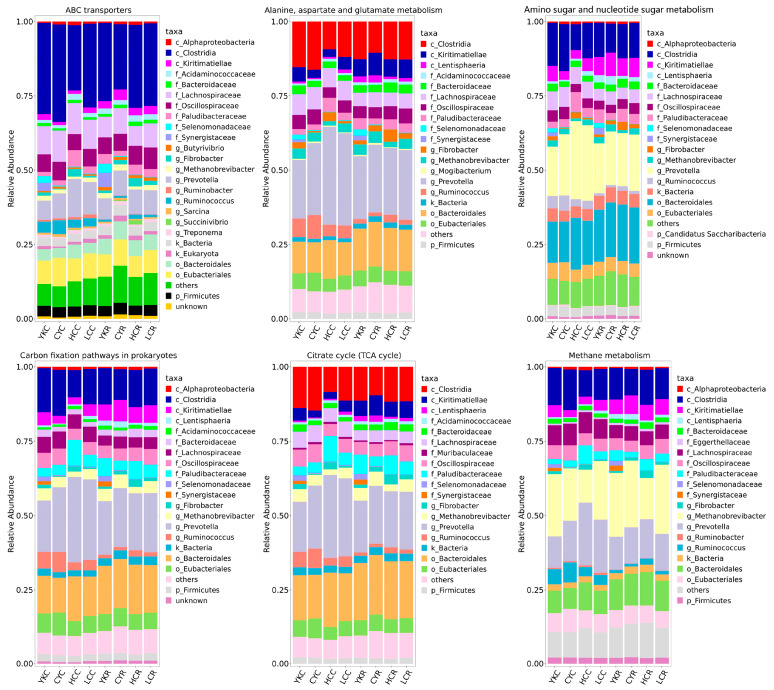
Relative contribution of different taxa to identified rumen-enriched functional attributes of KEGG pathway level 3 in 8 groups.

To visualize the association between rumen-enriched taxonomic and functional properties, this study identified the taxonomic origin of rumen-enriched functional attributes in eight groups. The top 15 rumen-enriched CAZymes at the family level (including GH13, CE0, GH5, CBM6, CBM48, GT2, etc.) and the top 15 identified KEGG pathways (including ABC transporters, alanine, aspartate, glutamate metabolism, amino sugar, nucleotide sugar metabolism, aminoacyl-tRNA biosynthesis, carbon fixation pathways in prokaryotes, citrate cycle (TCA cycle), pyruvate metabolism, methane metabolism, etc.) were conducted for the taxonomic and functional contribution analysis (Figure 9, Figure 10, Figures S1 and S2). With the exception of ABC transporters and methane metabolism, we found that *Prevotella* was the main contributor to almost all of these functions (Figure 10). Clostridia, *Prevotella*, *Fibrobacter* and Bacteroidales were major contributors to CBM6 and CBM48 (Figure 9). In CE0, the relative abundance of *Prevotella* and Bacteroidales was greater than 80% (Figure 8). Clostridia, *Prevotella* and Bacteroidales were also the largest contributors to GH13, GH5, GH97, GH31, GH2, GH10, GH43 and GH3 (Figure 9 and Figure S1). For the KEGG pathways at level 3, in methane metabolism, *Methanobrevibacter* had the highest relative abundance, followed by *Prevotella*, Clostridia and Bacteroidales in all groups. In ABC transporters, Clostridia was the main contributor, with more than 30% in eight groups, followed by *Prevotella*, Lachnospiraceae and Eubacteriales (Figure 10). Moreover, compared with C groups, the proportion of Clostridia decreased in YKR and CYR, increased in HCR, and remained unchanged in LCR. We also found that *Methanobrevibacter* was involved in several metabolic pathways, such as ABC transporters, alanine, aspartate and glutamate metabolism, aminoacyl-tRNA biosynthesis, amino sugar and nucleotide sugar metabolism, carbon fixation pathways in prokaryotes, citrate cycle (TCA cycle), etc. (Figure 10 and Figure S2). In addition, we also found that Selenomonadaceae and Synergistaceae were significantly observed in almost all YKR and YKC groups in top 15 KEGG pathways but were very low or absent in other groups (Figure 10 and Figure S2).

### 3.6. PCR Validation of Rumen Cellulase Genes

A total of 112 genes were verified by PCR amplification, of which 89 genes were successfully amplified and 23 genes were repeatedly amplified unsuccessfully, and the results are shown in Figure S3A–I.

## 4. Discussion

The main objective of this experiment was to study the effects of the feeding transition from all-concentrate to all-roughage on the patterns and functions of the rumen micro-organisms of yaks, cattle-yak, Tibetan cattle and common cattle using metagenomic sequencing. At the phylum level, Bacteroidetes and Firmicutes were dominant phyla in eight groups, which was similar to many studies [60,61,62]. Firmicutes included a variety of bacteria genera related to cellulose degradation, while Bacteroides, as Gram-negative bacteria, were mainly involved in the degradation of non-structural carbohydrates [63]. Generally, the relative abundances of Bacteroidetes and Firmicutes change with animal species and the conversion of concentrate and roughage. Firmicutes were found to dominate in high-grain feeds, while Bacteroidetes dominated in hay feeds [64]. In this experiment, it was also found that under roughage feeding conditions, the relative abundance of Bacteroidetes in rumen microflora of yak, cattle-yak, Tibetan yellow cattle and yellow cattle dominated and were higher than Firmicutes. After the conversion from concentrate feed to roughage, the abundance of Bacteroidetes increased in yak (32.83% to 38.98%) and cattle-yak (36.26% to 37.21%), especially in yaks. However, it decreased in both Tibetan yellow cattle (52.01% to 43.94%) and yellow cattle (42.91% to 41.54%). The proportion of Firmicutes in yak (48.57% to 38.31%) and cattle-yak (51.98% to 36.73%) decreased sharply, but the change was not significant in Tibetan yellow cattle (33.67% to 34.46%) and yellow cattle (40.47% to 37.86%). The above results show that compared with Tibetan yellow cattle and yellow cattle, yaks and cattle-yak can better adapt to the transition from all-concentrate to all-roughage, and the types and proportions of rumen microorganisms can be rapidly adjusted, which may be one of the important internal reasons why they are more conducive to adapting to the harsh plateau environment. This finding is also reflected in rumen fermentation parameters. From all-concentrate to all-roughage, other indexes except isobutyrate and valerate increased in yak and cattle-yak, especially the butyrate increase is significant. Compared with the C groups, the relative abundance of Lentisphaerae increased in rumen fluid in all R groups and was significantly increased in yak and cattle-yak but not significantly in Tibetan yellow cattle and yellow cattle. This may be because the Lentisphaerae mainly participate in the degradation of cellobiose [65], while yaks and cattle-yak may have stronger cellulose digestion ability and were better able to adapt to the harsh living environment in the plateau. In addition, compared with the C groups, the relative abundance of Kiritimatiellaeota in all R groups increased. It was found that Kiritimatiellaeota was mainly involved in fiber degradation, and the increase in the relative abundance of Kiritimatiellaeota in roughage groups was directly related to fiber content [66].

*Prevotella* belongs to Bacteroidetes and is mainly involved in the degradation of polysaccharides, proteins and starch in the rumen, but it cannot degrade polysaccharides in plant cellulose alone, and this process must cooperate with fibrilolytic bacteria [67,68]. In a study on the comparison of rumen microbiota structure and functional prediction among cattle-yak, yellow cattle and Jersey, it is found that the relative abundance of *Prevoella* in the rumen fluid of yellow cattle is higher than that of cattle-yak, indicating that yellow cattle may have more advantages in the utilization of non-cellulosic carbohydrates [69]. In this study, we also found that the relative abundance of *Prevoella* in the rumen of yellow cattle was higher than that of cattle-yak, and the relative abundance of Tibetan yellow cattle and yellow cattle decreased when the diet was changed from concentrate to roughage. As a common methanogenic bacteria, the relative abundance of *Methanobrevibacter* in the rumen fluid of yak, cattle-yak, Tibetan yellow cattle and yellow cattle increased when the diet changed from concentrate feed to roughage, which may be due to the symbiotic relationship between methanogens and cellulose decomposing bacteria [70]. *Ruminococcus* and *Ruminobacter* have strong amylase activity [71], and it has been observed that the relative abundance of *Ruminococcus* and *Ruminobacter* decreases in the four R groups in this experiment. *Butyrivibrio* belongs to anaerobic Gram-negative bacteria and is an important member of intestinal flora [72]. Its metabolites play an important role in the digestion and nutrient absorption of ruminants [73]. *Butyrivibrio* strain is capable of utilizing hydrogen and carbon dioxide produced in the rumen and converting sugars into butyric acid or other short-chain fatty acids through fermentation [74]. In rumen, butyric acid is the major fermentation product of *Butyrivibrio*, and short chain fatty acids such as butyric acid were not conducive to the growth and reproduction of methanogens, thus reducing methane production [75]. In this experiment, the relative abundance of *Butyrivibrio* in all R groups increased, and the change was significant in yaks, indicating that the growth and reproduction of methanogens in yaks were markedly inhibited, thus reducing the production and emission of methane, which was conducive to further improving the feed utilization rate of yaks.

When the diet was changed from concentrate to roughage feed, the content of non-structural carbohydrates decreased significantly, while the content of structural carbohydrates (such as cellulose, hemicellulose, lignin, etc.) increased significantly [76]. Cellulose and hemicellulose are the main structural components of plant cell wall, which are degraded and fermented by anaerobic microorganisms in rumen to produce volatile fatty acids and are the main nutritional sources of the host [26]. Cellulose degradation is mainly carried out by specialized bacteria but also by protists and fungi, through various mechanisms [26]. *Fibrobacter* is one of the cellulose-degrading bacteria, which can produce cellulose-degrading enzymes and hemicellulose-degrading enzymes. In this study, while the diet changed from concentrate to roughage feed, the relative abundance of *Fibrobacter* in cattle-yak, Tibetan yellow cattle and yellow cattle increased, significantly in cattle-yak and Tibetan yellow cattle, but not in yellow cattle. This indicated that roughage has a more significant effect on the relative abundance of *Fibrobacter* in cattle-yak and Tibetan yellow cattle. However, under the same conditions, the relative abundance of *Fibrobacter* in yaks decreased, but the difference was not significant, which may be related to the living environment of yaks. In the high cold environment, yaks mainly rely on limited herbs, lichens and other vegetation for food, and these plants grow slowly with high cellulose content, so yaks have a high tolerance to roughage [77]. In addition, due to the cold climate, the digestive system of yaks requires more energy to maintain body temperature and life activities, which may reduce the relative abundance of *Fibrobacter* [78]. *Lachnospiraceae bacterium*, a class of anaerobic Gram-positive bacteria, belonging to Bacteroideta, are one of the important members of intestinal microbiota, mainly involved in the degradation and metabolism of polysaccharides such as cellulose and hemicellulose [79]. However, in this study, the relative abundance of *Lachnospiraceae bacterium* in the four R groups all declined, which may be attributed to the fact that concentrate feed contains a large amount of protein and carbohydrates to provide rich nutrition, while roughage contains relatively high lignin or mineral content, which inhibits its growth and metabolism [18]. In the gastrointestinal tract of animals, Clostridiales bacterium are an important class of probiotics, which can decompose difficult-to-digest cellulose and hemicellulose, provide energy and nutrients, inhibit the growth of harmful bacteria, and maintain intestinal microecological balance [80]. Studies have shown that roughage can increase the abundance of clostridium bacteria and improve their ability to degrade polysaccharides [81], so as to increase the synthesis of short-chain fatty acids and the production of other beneficial metabolites, which is the same as the results of this experiment.

Different diets and animal species may affect the types and diversity of rumen microorganisms [82,83]. In this study, dietary change from concentrate to roughage increased the number of unique genes of rumen microorganisms in yak, cattle-yak, Tibetan yellow cattle and yellow cattle. The number of genes varied most in yaks and cattle-yaks but less in Tibetan yellow cattle and yellow cattle. Among them, the number of unique genes in CYR was the highest, and the difference between the concentrate and roughage group was the largest, while that in LCR was the lowest, and the difference between the concentrate and roughage group was the smallest. The conversion from concentrate to roughage had the greatest impact on cattle-yak, which may be related to its long-term living in an extreme environment in the plateau, stronger adaptability and higher utilization and conversion efficiency of roughage [84]. Therefore, the abundance of rumen microbial genes in cattle-yak was higher after the conversion from concentrate to roughage.

In four cattle groups, when concentrate was converted to roughage, the relative abundance of GH5, GH26, GH30, GH78, CE3, PL1, PL9 and CAZymes related to cellulose, hemicellulose and pectin degradation significantly increased, while GH13 and GH53 significantly decreased. Xylanase (such as GH5, GH26 and GH30) can digest the huge side chain of hemicellulose, exposing the backbone of lignin for xylanase hydrolysis [85,86]. However, this is not completely consistent with the change of xylanase activity measured in this study. This may be because xylanases are a large family rather than a single enzyme, which means that it contains many different enzymes [87]. These enzymes work together to degrade xylan, a heterogeneous polysaccharide found in plant cell walls, by working in concert with each other [88]. The biodegradation of xylanase requires the participation of many enzymes [88]. So, when companies measure xylanase activity in rumen fluid, they are actually measuring the overall activity of a group of enzymes, not a single enzyme. Pectin lyase (such as GH78, PL1 and PL9) can cut α-1, 4-glucoside bonds by β-trans elimination and degrade demethylated pectin to produce oligosaccharides [89,90]. This enzyme plays a key role in the metabolic network of plant cell walls. During roughage degradation, pectinase can decompose pectin in roughage, improve the degradation and utilization of cellulose in feed, and increase the digestibility of cellulose in animals [91]. GH13, which is a major α-amylase family, hydrolyzes the internal alpha-1, 4-glucoside bonds of starch-related carbohydrates [92]. Therefore, when the diet is changed from refined feed to roughage, the starch content in the diet is greatly reduced, so the abundance of starch in the four groups is decreased. The number of CAZymes encoded by rumen microbial functional genes is directly related to the diversity of rumen microbial structure and function. Conversion from concentrate to roughage significantly increased the number of CAZymes in all groups, indicating that roughage can increase the diversity of rumen microorganisms. In all groups, the members of the CBMs, GHs, GTs, CEs and PLs families with the highest relative abundance were CBM48, GH13, GT2, CE0 and PL1, respectively. The structural characteristics of CBM48 enable it to bind to glucose units in starch molecules, thus participating in the degradation and utilization of starch. CBM48 can also bind to starch molecules, locate starch molecules near amylase and promote the degradation of amylase [93]. GH13 is a family of glycosidases, including amylase, glucosidase, galactosidase, etc., which can catalyze the degradation and conversion of various carbohydrate substances, such as starch, glucose, galactose, etc. [94,95]. GT2 is a class of glycosyltransferase, which is mainly involved in starch synthesis but also plays a role in carbohydrate degradation [96,97]. CE0 can hydrolyze ester groups in starch, so as to promote starch degradation and utilization. In this study, the relative abundance of CBM48, GH13, GT2 and CE0 decreased after the diet was converted to roughage, which was consistent with many studies [98,99]. Pectin is a natural macromolecular compound belonging to structural carbohydrate, which is abundant in roughage (such as corn stalk), which is difficult to be degraded by intestinal microorganisms [100,101]. PL1 is a class of fructan hydrolase that promotes the hydrolysis of pectin into smaller monosaccharides and oligosaccharides, thereby providing energy and nutrients [102]. Therefore, in this study, the relative abundance of PL1 in group R was significantly higher than that in group C, which is similar to some results [103,104].

The metagenomic data obtained were functionally annotated, and the results showed that diet type and animal breed affected the content of rumen microorganisms, but the metabolic pathway was basically the same, and the main was metabolism. The main metabolic pathways included carbohydrate metabolism, amino acid metabolism, energy metabolism, etc. Methane metabolism can convert methane into useful products or purify and remove it, so as to better maintain the stability of the ecosystem and the sustainable development of human society [105]. Ruminants living in harsh environments at high altitudes have evolved individuals that produce high levels of volatile fatty acids and low levels of methane [17,22]. We found that the relative abundance of methane metabolism in the roughage group of yaks, cattle-yaks and Tibetan yellow cattle increased but decreased in yellow cattle. It is speculated that roughage will reduce the methane emissions of plateau ruminants and convert methane into energy for their own needs [106,107]. Butanoate metabolism is closely related to the methane production of ruminants [108]. A large number of microorganisms in the stomach of ruminants such as cattle and sheep can decompose nutrients to produce a large number of short-chain fatty acids, including butyrate [109]. In this study, it was found that acetate, propionate and butyrate in yaks and cattle-yaks all increased after the conversion from concentrate to roughage, and butyrate increased significantly, while they all decreased in Tibetan yellow cattle and yellow cattle, but the changes were not significant. For example, butyrate is a strong acidic substance, which easily reduces the pH of gastric juice and inhibits the growth of microorganisms [110]. Ruminant stomach contains a large number of methanogenic bacteria, which can grow and reproduce normally in a weakly acidic environment [111]. Therefore, the large accumulation of butyric acid in ruminant stomach will lead to a rapid decline in pH value, which will inhibit the growth and metabolism of methanogenic bacteria, thus reducing the production of methane in ruminants [112]. We also found that compared with the C groups, the relative abundance of butanoate metabolism in the yaks, cattle-yaks and Tibetan yellow cattle in the R groups decreased and that in cattle-yaks significantly decreased, while that in yellow cattle increased but not significantly. We conclude that from concentrate to roughage, yaks and cattle-yaks reduce rumen pH by increasing acetate, propionate and butyrate contents and reduce related metabolic pathways (butanoate metabolism, etc.) to inhibit the growth of methanogens, thus ultimately reducing methane emission. In addition, the relative abundance of citrate cycle in yaks, cattle-yaks, Tibetan yellow cattle and yellow cattle in the roughage group showed a significant decline, and the difference was significant in yaks. This may be related to the types of diets and the long-term living environment of animals. Soybean meal in concentrate groups is a high-protein with rich amino acid content, among which glutamic acid and aspartic acid can be converted into intermediate products of the citrate cycle, thus entering the citrate cycle for energy metabolism [113,114]. However, the content of carbohydrate and protein in roughage corn stalk is relatively low, which cannot directly provide enough intermediate metabolites to enter the citric acid cycle [115]. In summary, the conversion of concentrate to roughage can change the structure and diversity of rumen microorganisms and may reduce the methane production in yaks, cattle-yaks and Tibetan yellow cattle living in the plateau while increasing the methane production in yellow cattle, which can more effectively improve the utilization rate of roughage for ruminants on the plateau.

## 5. Conclusions

This study described the effects of conversion from all-concentrate to all-roughage diets on the ruminal fermentation parameters, rumen microbiota pattern and function of plateau in high-altitude yaks, cattle-yaks, Tibetan yellow cattle and low-altitude yellow cattle by metagenomic sequencing. In fact, the transition mode of all-concentrate to all-roughage diets altered the ruminal fermentation pattern and the structure and composition of the microflora of yaks, cattle-yaks, Tibetan yellow cattle and yellow cattle, which in turn affected their functions, but the effects on yaks and cattle-yaks were more obvious. Acetate, propionate and butyrate in yaks and cattle-yaks all increased after the conversion from concentrate to roughage, and butyrate increased significantly, while they all decreased in Tibetan yellow cattle and yellow cattle, but the changes were not significant. The extreme dietary conversion increased the richness and diversity of the rumen microbiota in four herds, but Bacteroidetes and Firmicutes were still dominant in all treatment groups at the phylum level, and the changes were most obvious in the yaks and cattle-yaks. At the genus and species levels, *Prevotella* has the highest abundance, and with the exception of ABC transporters and methane metabolism, we found that *Prevotella* is a major contributor to almost all of these functions. Compared with the concentrate diets, the relative abundance of citric acid cycle in roughage groups decreased, especially in the yaks. The relative abundancy of butanoate metabolism in the yaks, cattle-yaks and Tibetan yellow cattle decreased, while that in the yellow cattle increased. We concluded that after the conversion from all-concentrate to all-roughage, the methane production of the yaks, cattle-yaks and Tibetan yellow cattle would decrease, among which the yaks and cattle-yaks were the most obvious. Yaks and cattle-yaks may appropriately reduce rumen pH by increasing the production of acetic acid, propionic acid and butyric acid, thereby inhibiting the growth of methanobacteria, which will ultimately reduce methane emissions. However, the metabolic pattern of the yellow cattle was the opposite. In conclusion, the crude feeding tolerance of the yaks, cattle-yaks and Tibetan yellow cattle is stronger, but the utilization rate of crude feeding of the yaks and cattle-yaks is higher and more environmentally friendly, which is more suitable for intensive raising of livestock on the plateau. The above research provides a theoretical basis for the intensive feeding of livestock in the plateau.

## Figures and Tables

**Table 1:** Experimental groups and sample information.

Groups	Concentrate Groups (*n* = 6)	Roughage Groups (*n* = 6)
Yak (YK)	YKM-1/YKM-2/YKM-3/ YKF-4/YKF-5/YKF-6	YKM-11/YKM-12/YKM-13/ YKF-14/YKF-15/YKF-16
Cattle-yak (CY)	CYM-1/CYM-2/CYM-3 /CYF-4/CYF-5/CYF-6	CYM-11/CYM-12/CYM-13/ CYF-14/CYF-15/CYF-16
High-altitude cattle (HC)	HCM-1/HCM-2/HCM-3 /HCF-4/HCF-5/HCF-6	HCM-11/HCM-12/HCM-13 /HCF-14/HCF-15/HCF-16
Low-altitude cattle (LC)	LCM-1/LCM-2/LCM-3 /LCF-4/LCF-5/LCF-6	LCM-11/LCM-12/LCM-13 /LCF-14/LCF-15/LCF-16

Note: High-altitude cattle represents the Tibetan yellow cattle; Low-altitude cattle represents yellow cattle; M: Male and F: Female, for example, YKM-1 represents a male yak numbered 1 and YKF-4 represents a female yak numbered 4.

**Table 2:** Rumen carbohydrate enzyme activities of yaks, cattle-yak, Tibetan yellow cattle and yellow cattle fed with concentrate to roughage diets.

Groups	Items	Treatments ^1^		SEM ^2^	*p* Value
		C	R		
YK	Xylanase activity	0.152	0.153	0.014	0.456
	Cellobiase activity	0.202 ^b^	0.170 ^a^	0.012	<0.05
	Microcrystalline cellulase activity	0.173	0.162	0.009	0.136
	Carboxymethyl cellulase activity	0.190	0.193	0.014	0.413
CY	Xylanase activity	0.165	0.142	0.014	0.079
	Cellobiase activity	0.207 ^b^	0.172 ^a^	0.011	<0.05
	Microcrystalline cellulase activity	0.190 ^b^	0.160 ^a^	0.011	<0.05
	Carboxymethyl cellulase activity	0.210 ^B^	0.173 ^A^	0.008	<0.01
LC	Xylanase activity	0.153	0.142	0.012	0.182
	Cellobiase activity	0.192 ^b^	0.167 ^a^	0.008	<0.05
	Microcrystalline cellulase activity	0.163 ^b^	0.152 ^a^	0.004	<0.05
	Carboxymethyl cellulase activity	0.195	0.185	0.012	0.224
HC	Xylanase activity	0.145 ^b^	0.128 ^a^	0.006	<0.05
	Cellobiase activity	0.177 ^B^	0.150 ^A^	0.002	<0.01
	Microcrystalline cellulase activity	0.158 ^B^	0.138 ^A^	0.004	<0.01
	Carboxymethyl cellulase activity	0.198	0.177	0.013	0.082

Note: Peer data with no letter shoulder indicate no significant difference (*p* > 0.05), peer data with lowercase letter shoulder indicate significant difference (*p* < 0.05) and peer data with uppercase letter shoulder indicate very significant difference (*p* < 0.01). ^1^ Treatments: C (concentrated diets); R (roughage diets). ^2^ SEM: standard error of the mean.

**Table 3:** Rumen fermentation profile of yaks, cattle-yak, Tibetan yellow cattle and yellow cattle fed with concentrate and roughage diets.

Groups	Parameters (mmol/L)	Treatments ^1^		SEM ^2^	*p* Value
		C	R		
YK	Acetate	31.417	37.950	6.286	0.173
	Propionate	7.718	8.577	1.614	0.309
	Isobutyrate	0.539	0.444	0.082	0.149
	Butyrate	3.160 ^b^	5.022 ^a^	0.837	<0.05
	Isovalerate	0.656	0.692	0.167	0.418
	Valerate	0.397	0.320	0.073	0.170
	NH3-N	4.503	4.782	0.785	0.330
CY	Acetate	33.667	35.300	2.972	0.303
	Propionate	7.442	8.770	1.240	0.167
	Isobutyrate	0.586	0.483	0.052	0.052
	Butyrate	2.938 ^b^	4.520 ^a^	0.529	<0.05
	Isovalerate	0.639	0.662	0.108	0.421
	Valerate	0.361	0.340	0.081	0.401
	NH3-N	3.987	5.135	1.574	0.249
LC	Acetate	32.167	30.500	2.185	0.240
	Propionate	7.005	6.490	0.587	0.210
	Isobutyrate	0.418	0.415	0.035	0.464
	Butyrate	3.603	3.567	0.381	0.464
	Isovalerate	0.535	0.573	0.076	0.321
	Valerate	0.327	0.243	0.044	0.057
	NH3-N	2.620 ^b^	4.293 ^a^	0.543	<0.05
HC	Acetate	29.407	29.404	1.630	0.500
	Propionate	7.358	7.295	0.648	0.463
	Isobutyrate	0.434 ^b^	0.355 ^a^	0.036	<0.05
	Butyrate	2.422	2.860	0.513	0.216
	Isovalerate	0.524 ^b^	0.428 ^a^	0.042	<0.05
	Valerate	0.252	0.189	0.032	0.053
	NH3-N	1.798	1.773	0.315	0.470

Note: Peer data with no letter shoulder indicate no significant difference (*p* > 0.05), peer data with lowercase letter shoulder indicate significant difference (*p* < 0.05). ^1^ Treatments: C (concentrated diets); R (roughage diets). ^2^ SEM: standard error of the mean.

**Table 4:** Comparison of number of unique genes between concentrate and roughage groups.

Groups	Common	Unique in C	Unique in R	Total
YKR vs. YKC	11,879	373	943	13,195
CYR vs. CYC	11,847	245	1149	13,241
HCR vs. HCC	11,548	399	917	12,864
LCR vs. LCC	11,908	404	754	13,066

Note: The number of genes in each group is not a direct average of the number of genes in the 6 individuals but a calculated union of genes in the 6 individuals. As far as genes are concerned, there are differences in the number of genes and the names of genes in each group; it is more biologically significant to use union than direct average.

## Data Availability

All raw and processed sequencing data generated in this study have been submitted to the NCBI, metagenomic data (yak, yellow cattle, Tibetan yellow cattle and cattle-yak) with BioProject accession PRJNA1070712 (https://dataview.ncbi.nlm.nih.gov/object/PRJNA1070712?reviewer=pgkvr1obgu40nff9ujba3a1ddk, accessed on 30 January 2024); PRJNA1070714 (https://dataview.ncbi.nlm.nih.gov/object/PRJNA1070714?Reviewer=ir3mv8gbulrhaapsli7noejb2r, accessed on 30 January 2024); PRJNA1070716 (https://dataview.ncbi.nlm.nih.gov/object/PRJNA1070716?reviewer=p3oc6ui9qp7m68h95usb01ncsh, accessed on 30 January 2024); PRJNA1070718 (https://dataview.ncbi.nlm.nih.gov/object/PRJNA1070718?reviewer=sifue1dub13d9b2racoc4ir3gu, accessed on 30 January 2024).

## Supplementary Materials

The following supporting information can be downloaded at https://www.mdpi.com/article/10.3390/ani14202933/s1. Table S1: Design of primers for PCR validation of cellulase genes; Table S2: Statistical table of quality control results; Table S3: Rumen microbial abundance of 8 groups (YKR, YKC, CYR, CYC, HCR, HCC, LCR and LCC) at different levels; Table S4: Rumen microbial abundance of 2 groups (R and C) at different levels; Table S5: Rumen microbial abundance table at genus level; Table S6: The number of functional genes encoding different CAZymes in the concentrate and roughage groups; Table S7: The relative abundance of all CAZymes in the concentrate and roughage groups. Figure S1: Relative contribution of different taxa to identified rumen-enriched functional attributes of CAZymes encoded genes at the family level in different samples; Figure S2: Relative contribution of different taxa to identified rumen-enriched functional attributes of KEGG pathway level 3 in different samples. Figure S3: PCR validation of rumen cellulase genes.

## Abbreviations

DEGs	Differentially expressed genes
GHs	Glycoside hydrolases
GTs	Glycosyltransferases
CBMs	Carbohydrate-binding modules
CEs	Carbohydrate esterases
PLs	Polysaccharide lyases
AAs	Auxiliary activities
KEGG	Kyoto Encyclopedia of Genes and Genomes
CAZy	Carbohydrate-active Enzymes
