# Supplementary material for: Dietary Conversion from All-Concentrate to All-Roughage Alters Rumen Bacterial Community Composition and Function in Yak, Cattle-Yak, Tibetan Yellow Cattle and Yellow Cattle
Source: Animals (Basel). 2024 Oct 11;14(20):2933. doi: 10.3390/ani14202933 (PMC11503692; doi:10.3390/ani14202933)
Supplement: Supplementary file 1 [file animals-14-02933-s001.zip › Figure S3-PCR validation of rumen cellulase genes.pdf]

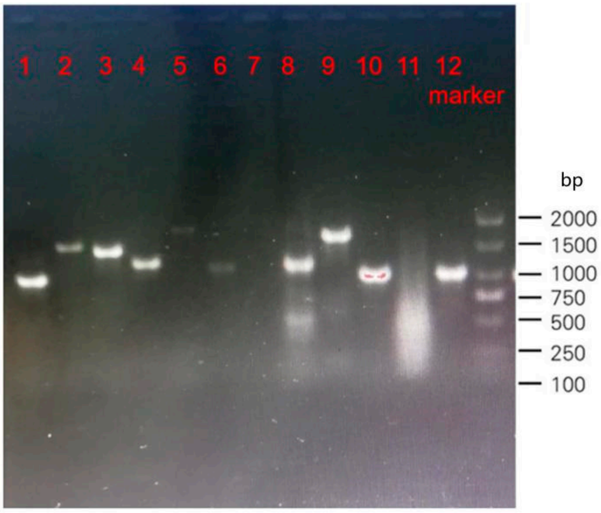

Figure S3A. PCR results from Deeplab-GH10-1 to Deeplab-GH10-12. Line7 and line 11 have no amplified strips.

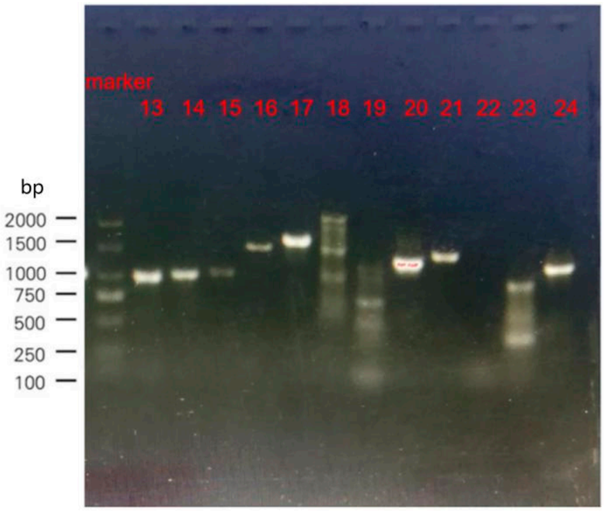

Figure S3B. PCR results from Deeplab-GH10-13 to Deeplab-GH10-24.Line22 have no amplified strip.

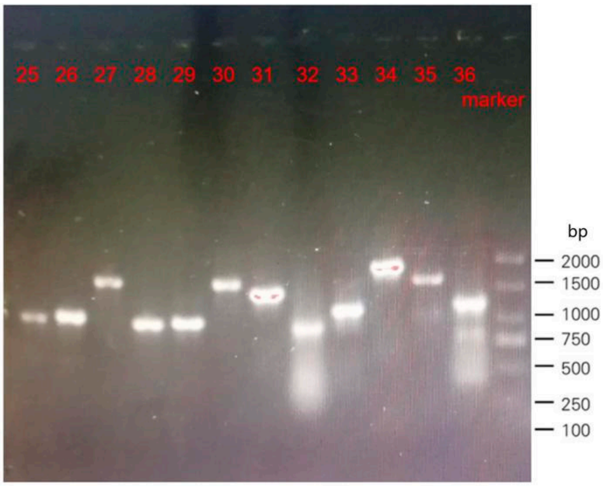

Figure S3C. PCR results from Deeplab-GH10-25 to Deeplab-GH10-36

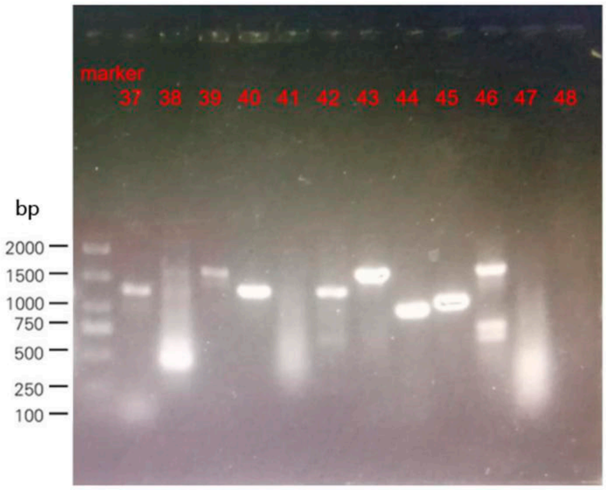

Figure S3D. PCR results from Deeplab-GH10-37 to Deeplab-GH10-48. Line38、41、47 and 48 have no amplified strips.

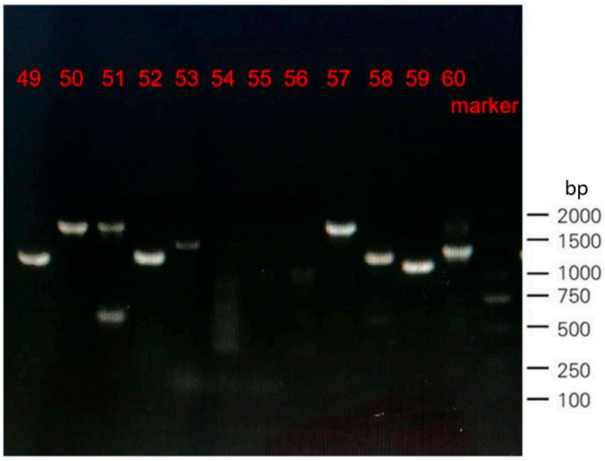

Figure S3E PCR results from Deeplab-GH10-49 to Deeplab-GH10-60. Line54 and 55 have no amplified strips.

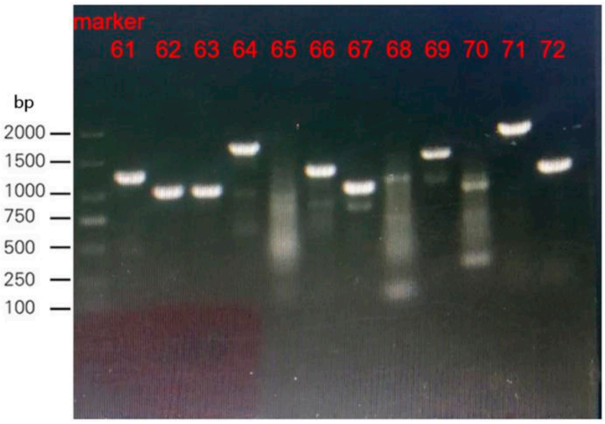

Figure S3F PCR results from Deeplab-GH10-61 to Deeplab-GH10-72. Line65 have no amplified strips.

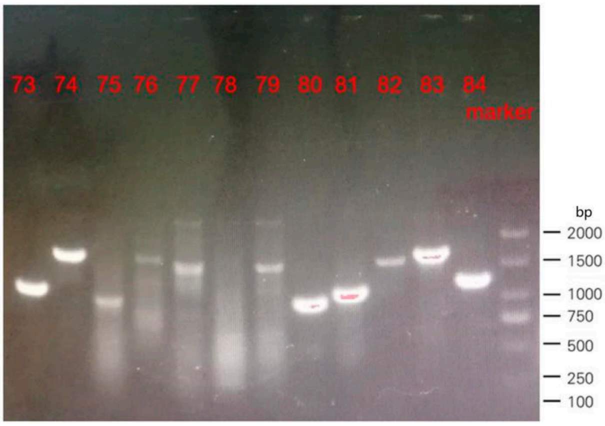

Figure S3G. PCR results from Deeplab-GH10-73 to Deeplab-GH10-84.

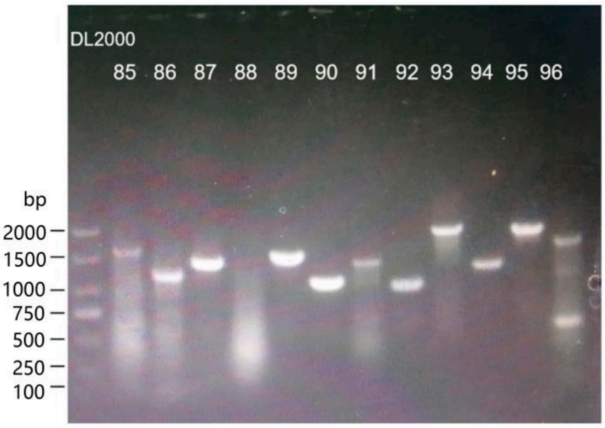

Figure S3H. PCR results from Deeplab-GH10-85 to Deeplab-GH10-96. Line 88 have no amplified strips.

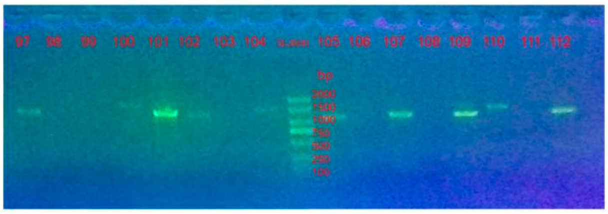

Figure S3I. PCR results from Deeplab-GH10-97 to Deeplab-GH10-112. Line 98、99、103、106、108 and 111 have no amplified strips.
