# Supplementary material for: Dietary Conversion from All-Concentrate to All-Roughage Alters Rumen Bacterial Community Composition and Function in Yak, Cattle-Yak, Tibetan Yellow Cattle and Yellow Cattle
Source: Animals (Basel). 2024 Oct 11;14(20):2933. doi: 10.3390/ani14202933 (PMC11503692; doi:10.3390/ani14202933)
Supplement: Supplementary file 1 [file animals-14-02933-s001.zip › Table S1-Design of primers for PCR validation of cellulase genes.pdf]

Table S1 Design of primers for PCR validation of cellulase genes

Serial number	Gene number	Primer name	Primer sequence
1	CYF-04_408835_1	Deeplab-GH10-1-F	ATGAACGAAGTCTTTTCCCTGGC
	CYF-04_408835_1	Deeplab-GH10-1-R	TTATTCCTTTACCGCATCCAGCAG
2	CYF-04_716392_20	Deeplab-GH10-2-F	ATGAAAACGAAGATGATTGTGGTCGC
	CYF-04_716392_20	Deeplab-GH10-2-R	TCACTTCAGCTCGACGAGCTTC
3	CYF-05_42567_2	Deeplab-GH10-3-F	ATGATAAAAAAAATAACCATCATTTGTGG
	CYF-05_42567_2	Deeplab-GH10-3-R	TTACTTGTATACATTGATGATAGAGTCG
4	CYF-05_48674_2	Deeplab-GH10-4-F	ATGAGATCTAAGTTAAAACAACTATTATTATTGG
	CYF-05_48674_2	Deeplab-GH10-4-R	TTAATAAGGATACTTTGCTGCAGCG
5	CYF-06_56150_2	Deeplab-GH10-5-F	ATGAAGATGGCTTCGTGTGTAGAC
	CYF-06_56150_2	Deeplab-GH10-5-R	TCATTCACCGCTCAGACCGTC
6	CYF-06_1094850_1	Deeplab-GH10-6-F	ATGAAAAGTAAATTGTTTGTTTTAGC
	CYF-06_1094850_1	Deeplab-GH10-6-R	TCATTTGAACAGTTTGATAATCTTC
7	CYF-14_94727_1	Deeplab-GH10-7-F	ATGATGAAAAAAGCTCTCCTTATGCTC
	CYF-14_94727_1	Deeplab-GH10-7-R	TTACGGCATTTTGCCGGTTTTGG
8	CYF-14_142257_2	Deeplab-GH10-8-F	ATGAAAAAGCCAATTGCCCTTGTTGC
	CYF-14_142257_2	Deeplab-GH10-8-R	TCATTTCAGCTCCACTAGTTTAGTG
9	CYF-15_1277055_10	Deeplab-GH10-9-F	ATGAAAAAAGGTTGTATACGTGCGG
	CYF-15_1277055_10	Deeplab-GH10-9-R	TCATTTCACGTCGACCACCTTG
10	CYF-15_1843574_2	Deeplab-GH10-10-F	ATGAAAAAGCTACTCTTCTTTCTTC
	CYF-15_1843574_2	Deeplab-GH10-10-R	CTATTTTCCGACAAGACGCC
11	CYF-16_464236_13	Deeplab-GH10-11_F	ATGAAAGCAGCATTGTCGGTG
	CYF-16_464236_13	Deeplab-GH10-11-R	TCAGCGGAGTTCGACGAG
12	CYF-16_481517_4	Deeplab-GH10-12-F	ATGAAAAAAGGTACACTTTTACTTCTC
	CYF-16_481517_4	Deeplab-GH10-12-R	TTACTTCCCGACGAGGCGG
13	CYM-01_1144364_2	Deeplab-GH10-13-F	ATGAAAAAAATTCTGTTAGCGCTGCTG
	CYM-01_1144364_2	Deeplab-GH10-13-R	TCATCTCACAACCCATTTACGGC
14	CYM-01_1223842_3	Deeplab-GH10-14-F	ATGAAGAAAATCAGTCTTGCAGTG
	CYM-01_1223842_3	Deeplab-GH10-14-R	CTATTTCTTTTTATTGGCCGCGG
15	CYM-03_679495_2	Deeplab-GH10-15-F	ATGAAAAAATTTGAAGGATATACCCAC
	CYM-03_679495_2	Deeplab-GH10-15-R	TTATCTGAAATCGGGACTGATTTC
16	CYM-03_1912288_2	Deeplab-GH10-16-F	ATGGATTTCATCAAGGAGTGCTAC
	CYM-03_1912288_2	Deeplab-GH10-16-R	TCATTCCAATGTAACGTTTTCTGTGC
17	CYM-11_382789_11	Deeplab-GH10-17-F	ATGAAGAAATTCATTTGGTTGTCACTC
	CYM-11_382789_11	Deeplab-GH10-17-R	TTATTTATGAATATATTTCTTTCCGTTGC
18	CYM-11_848734_2	Deeplab-GH10-18-F	ATGAGAATTAAATTACTATTTTTATTCTTCC
	CYM-11_848734_2	Deeplab-GH10-18-R	TTAAGCACCTGCAACTTGC
19	CYM-12_449469_2	Deeplab-GH10-19-F	ATGAAGATCTTATTGAGTTTGACGATG
	CYM-12_449469_2	Deeplab-GH10-19-R	TTAGTCCAATTTGGGCTTTACGTTC
20	CYM-12_682308_2	Deeplab-GH10-20-F	ATGAAAATCAGTAAATATTTTATTCCTGC
	CYM-12_682308_2	Deeplab-GH10-20-R	TTAATGAATAGAAATCCAACTTAACATC
21	CYM-13_202408_25	Deeplab-GH10-21-F	ATGATGAATATGCAGAGATATGAAC
	CYM-13_202408_25	Deeplab-GH10-21-R	TCAATGAAGATTGATTTCGACTGTTTC
22	CYM-13_1644987_1	Deeplab-GH10-22-F	ATGAAAGTGAATATATATTTATTGCTTTTGATATGC
	CYM-13_1644987_1	Deeplab-GH10-22-R	TTATTGTCCAGCAACTTGCATAACAG
23	HCF-04_1084824_5	Deeplab-GH10-23-F	ATGGCCCAAGAAGGGAAGGGAC

	HCF-04_1084824_5	Deeplab-GH10-23-R	TTACTTGAAGTCCCTGACCACG
24	HCF-04_1087305_1	Deeplab-GH10-24-F	ATGTCCTACGAGCATCGCAAAG
	HCF-04_1087305_1	Deeplab-GH10-24-R	TTACAGCGTGACCTCCAGCTC
25	HCF-05_184169_2	Deeplab-GH10-25-F	ATGAAAAAGATTCTGATGTTCGCTATGG
	HCF-05_184169_2	Deeplab-GH10-25-R	TTATTCGATCTGTTCCGGATCCG
26	HCF-05_489252_4	Deeplab-GH10-26-F	ATGAAAAAGATATTCCTTCTCACCGC
	HCF-05_489252_4	Deeplab-GH10-26-R	TTATTTCTCCTGCTCGAAGAGGC
27	HCF-06_142817_7	Deeplab-GH10-27-F	ATGGTAAAAAAATCGCTTTATCTGTTGG
	HCF-06_142817_7	Deeplab-GH10-27-R	TTAGTTCGTTTTCCCACTCAGTGC
28	HCF-06_1500174_7	Deeplab-GH10-28-F	ATGAACAAAATTCTAATATCAGCACTTTGTG
	HCF-06_1500174_7	Deeplab-GH10-28-R	AAATCCCGGATAACGTAATACGCC
29	HCF-14_580639_2	Deeplab-GH10-29-F	ATGACAAGCAAAGTGAATCAGTCAAG
	HCF-14_580639_2	Deeplab-GH10-29-R	TCAATAACGCCTGAGCAGTTCG
30	HCF-14_1051565_12	Deeplab-GH10-30-F	ATGATTACCAAGATTTATAATAAGGTAGCG
	HCF-14_1051565_12	Deeplab-GH10-30-R	TTACTGTCCGCTGAGGGCG
31	HCF-15_1425263_42	Deeplab-GH10-31-F	ATGAAAAAACTGCTTTTATCAATTGCCG
	HCF-15_1425263_42	Deeplab-GH10-31-R	TCACTTGACCGTAAACTCTTTACTC
32	HCF-15_1571353_6	Deeplab-GH10-32-F	ATGCGAAGAAATAAACTTACACCGATC
	HCF-15_1571353_6	Deeplab-GH10-32-R	TTATTGGTCGTACCCGGCGTG
33	HCF-16_107257_8	Deeplab-GH10-33-F	ATGTCTTACGAGCATCGCAAAGC
	HCF-16_107257_8	Deeplab-GH10-33-R	TTATTCCAGCGTGATTTCGATGTCC
34	HCF-16_814871_9	Deeplab-GH10-34-F	ATGAAGAATTTCAAAATTACTGCCACTG
	HCF-16_814871_9	Deeplab-GH10-34-R	TTACTGAAGAAGTGAATTAAGCTTTTCAG
35	HCM-01_114503_2	Deeplab-GH10-35-F	ATGAGAAACAGAATGAAGATGGCGG
	HCM-01_114503_2	Deeplab-GH10-35-R	CTACAGCTCCACAAGTTTGGAAAC
36	HCM-01_1235332_75	Deeplab-GH10-36-F	ATGGATAAATATCAGCACAGAAAAGC
	HCM-01_1235332_75	Deeplab-GH10-36-R	TTACAGGATTATCGGCTGTTCGG
37	HCM-02_12479_2	Deeplab-GH10-37-F	ATGCTCGTCATGCCTGTGG
	HCM-02_12479_2	Deeplab-GH10-37-R	TCAGTATTTGGGTATAGTTGATGTAAAC
38	HCM-02_590236_11	Deeplab-GH10-38-F	ATGAAAAAGATTTTTAGAAACGGTCTC
	HCM-02_590236_11	Deeplab-GH10-38-R	TTAATTTCCGCTGAGGGCATCG
39	HCM-03_403538_1	Deeplab-GH10-39-F	ATGAAAGTCAAAAAAATATATTTTTTATTTTTTATC
	HCM-03_403538_1	Deeplab-GH10-39-R	TTACCCTAGATATTTAATTTCGAATTTAG
40	HCM-03_1020217_4	Deeplab-GH10-40-F	ATGAAAAAACTATTAACCTTAGCCG
	HCM-03_1020217_4	Deeplab-GH10-40-R	TTATTCCAAAGCTTTAATTACTTTAAAG
41	HCM-11_232583_3	Deeplab-GH10-41-F	ATGGAAGACCCCCTTTCCCAAG
	HCM-11_232583_3	Deeplab-GH10-41-R	CTAGAGCACCCGCGCGG
42	HCM-11_1034088_3	Deeplab-GH10-42-F	ATGAAAAAACTTTCAACAATCGCTCTC
	HCM-11_1034088_3	Deeplab-GH10-42-R	TTACTTCTTGAAGTCGCGAACAG
43	HCM-12_654655_2	Deeplab-GH10-43-F	ATGAGACTGTTATTGAGTTTGACAATG
	HCM-12_654655_2	Deeplab-GH10-43-R	TTATTCGAGTTTCGGCTTTACGTTC
44	HCM-12_1025564_1	Deeplab-GH10-44-F	ATGAACAAGAATATAACTATTATACTTTTATTTTTCG
	HCM-12_1025564_1	Deeplab-GH10-44-R	CTATTGGCCAGCAACTTGCATAATAG
45	HCM-13_35525_65	Deeplab-GH10-45-F	ATGGCAATAAAGAATAGAATCATCATATACTG
	HCM-13_35525_65	Deeplab-GH10-45-R	TTATTGCTGTTGATAGAGTTTGATAATGTC
46	HCM-13_175359_6	Deeplab-GH10-46-F	ATGAGAAACAGAATGGCGATGGC

	HCM-13_175359_6	Deeplab-GH10-46-R	TCACTTTACCTCAACCATCTTCGTC
47	LCF-04_250025_2	Deeplab-GH10-47-F	ATGAGCGAGCCGCTCCGC
	LCF-04_250025_2	Deeplab-GH10-47-R	CTAGGGCCGCGACGGCG
48	LCF-04_538680_1	Deeplab-GH10-48-F	ATGGACACCCCGTTTTCCCAAG
	LCF-04_538680_1	Deeplab-GH10-48-R	TCAGCCGACGCGCGCGTC
49	LCF-05_586399_8	Deeplab-GH10-49-F	ATGAAAAAACTATTCTTAGCAAGTATCATAGC
	LCF-05_586399_8	Deeplab-GH10-49-R	TTATTTCTTTTTCTTTGTCTTCTTAGCAGG
50	LCF-05_963461_1	Deeplab-GH10-50-F	ATGAATACATTATTTAGAAACATATCTCTG
	LCF-05_963461_1	Deeplab-GH10-50-R	TTACTTTCCACTCAGTGCATC
51	LCF-06_1031634_21	Deeplab-GH10-51-F	ATGGTAAAAAAATCGCTCTATCTATTGG
	LCF-06_1031634_21	Deeplab-GH10-51-R	TTATTTTCCACTGAGGGCATTGCAG
52	LCF-06_1255028_4	Deeplab-GH10-52-F	ATGTCCTACGATCATCGCAAGG
	LCF-06_1255028_4	Deeplab-GH10-52-R	TTACAGAATCAGGTCGATGTCCTTTC
53	LCF-14_385157_5	Deeplab-GH10-53-F	ATGAAACGTTTGCTTTTTTTCTTTTTTCTTTC
	LCF-14_385157_5	Deeplab-GH10-53-R	TTAATGCACCTGGACCCGTTTC
54	LCF-14_1276281_2	Deeplab-GH10-54-F	ATGTCATACGAACACCGCAAGG
	LCF-14_1276281_2	Deeplab-GH10-54-R	CTATTTCGACAGGCAGCCCTTC
55	LCF-15_251508_2	Deeplab-GH10-55-F	ATGAATGAGTTTGCAAACAAAATTCTGATTC
	LCF-15_251508_2	Deeplab-GH10-55-R	TTACCTGACGGAAAGCCGAATC
56	LCF-15_1538336_3	Deeplab-GH10-56-F	ATGAACGCAGAAAAGATAACGGCC
	LCF-15_1538336_3	Deeplab-GH10-56-R	TTAGACGGTCGCTTTCGTAGTC
57	LCF-16_523386_10	Deeplab-GH10-57-F	ATGAAGCAACTTTATATAGTTCTTTTTGC
	LCF-16_523386_10	Deeplab-GH10-57-R	TCACTTAATTACGACCTTTTTTCC
58	LCF-16_761797_2	Deeplab-GH10-58-F	ATGTCTCGGGATCCCTTCACTC
	LCF-16_761797_2	Deeplab-GH10-58-R	TTATTCGGCCAGGTGCAGAACG
59	LCM-01_411049_4	Deeplab-GH10-59-F	ATGAAAAAATTCCTTTTCCTATCAGTC
	LCM-01_411049_4	Deeplab-GH10-59-R	TTACATCAGTCCCTTCTGCTTC
60	LCM-01_1263646_4	Deeplab-GH10-60-F	ATGAAGGATCTCCTTGACCATGTC
	LCM-01_1263646_4	Deeplab-GH10-60-R	TTAATTTCCACTTAGCGCATCGCAG
61	LCM-02_312248_93	Deeplab-GH10-61-F	ATGAAAAAGAGTTGCAGAATCCTGG
	LCM-02_312248_93	Deeplab-GH10-61-R	TCAATTCTTCTCCGCGTATGATTTC
62	LCM-02_480460_10	Deeplab-GH10-62-F	ATGAAAAAGCTGTTCCTCCTTCTCC
	LCM-02_480460_10	Deeplab-GH10-62-R	TCAGTCTTTCCCCACGATTCTG
63	LCM-03_144345_1	Deeplab-GH10-63-F	ATGAAAAAGTTCCTTATCCTTCTCC
	LCM-03_144345_1	Deeplab-GH10-63-R	TCACTCCTTCCCGACGAGG
64	LCM-03_685039_6	Deeplab-GH10-64-F	ATGAAGAAGATTTTTAGAAACTTTACTC
	LCM-03_685039_6	Deeplab-GH10-64-R	TTAATTTCCACTTAGCGCATCAC
65	LCM-11_243510_2	Deeplab-GH10-65-F	ATGAAGAAGATTGTTGCTACCGCC
	LCM-11_243510_2	Deeplab-GH10-65-R	TCACTCGACCTCGACGAGC
66	LCM-11_1213698_4	Deeplab-GH10-66-F	ATGGGTAGATTTGAACACAGAAAAGC
	LCM-11_1213698_4	Deeplab-GH10-66-R	TTAAATTATTTTTTCAACGATCGCGTCAG
67	LCM-12_466764_7	Deeplab-GH10-67-F	ATGAAAAAGAAAGTCACCGCAATTGTC
	LCM-12_466764_7	Deeplab-GH10-67-R	CTAATCTTCTGGCGGAACAAGC
68	LCM-12_1110995_3	Deeplab-GH10-68-F	ATGCCCTCCCCGCTCTCC
	LCM-12_1110995_3	Deeplab-GH10-68-R	CTAGATGGATGTCGTCGCTTCG
69	LCM-13_878708_3	Deeplab-GH10-69-F	ATGAAGACATTTAAATACATGCTATTAGC

	LCM-13_878708_3	Deeplab-GH10-69-R	TTAATAGTCATTTGCCCTCAGAAGG
70	LCM-13_1316477_2	Deeplab-GH10-70-F	ATGAATTCTTGGAAGCATCGAAAAGC
	LCM-13_1316477_2	Deeplab-GH10-70-R	TCAGAGGAAGGGGTCTTCCG
71	YKF-04_77009_18	Deeplab-GH10-71-F	ATGAAGCACGTGAGTTTTATAGTGG
	YKF-04_77009_18	Deeplab-GH10-71-R	TCACTTGACCATGGTGTGTTGC
72	YKF-04_148377_6	Deeplab-GH10-72-F	ATGAAAAAATTCCTTTTCCTGTCAGTC
	YKF-04_148377_6	Deeplab-GH10-72-R	TTACATGAGTCCCTTCTTCTTCATG
73	YKF-05_30773_14	Deeplab-GH10-73-F	ATGAAACACATAATCTCTCTCTCAATAC
	YKF-05_30773_14	Deeplab-GH10-73-R	TTATTCGAAGATTTTAATCACCTGTTGG
74	YKF-05_139057_26	Deeplab-GH10-74-F	ATGAATAAGATTTTTAGAAACTTATCTC TG
	YKF-05_139057_26	Deeplab-GH10-74-R	TTATTGTCCACTAAGTGCCTTGC
75	YKF-06_274879_2	Deeplab-GH10-75-F	ATGAAGAGATGGAAAGGCTATGAGC
	YKF-06_274879_2	Deeplab-GH10-75-R	TTACAGGAGGGCGATGATCTC
76	YKF-06_1463011_7	Deeplab-GH10-76-F	ATGATTACAAAGATATTTAATAAGGTA GCGC
	YKF-06_1463011_7	Deeplab-GH10-76-R	TTACTGTCCTTTTAGGGCATCGC
77	YKF-14_806772_7	Deeplab-GH10-77-F	ATGAAAGGCCACGAAATGAAGACG
	YKF-14_806772_7	Deeplab-GH10-77-R	TCATTCCACCTCGACGAACTTG
78	YKF-14_1261071_2	Deeplab-GH10-78-F	ATGAACAAGATGCAGATGATGGCC
	YKF-14_1261071_2	Deeplab-GH10-78-R	CTACAGCAGCTGGCAGGC
79	YKF-15_251440_6	Deeplab-GH10-79-F	ATGAAACTGCTTCTGATTGCTGCC
	YKF-15_251440_6	Deeplab-GH10-79-R	CTATTCCAAGTCCACGAGCTTTTC
80	YKF-15_741360_11	Deeplab-GH10-80-F	ATGCGTGGTATGGAAGGCTATATG
	YKF-15_741360_11	Deeplab-GH10-80-R	TTAAAGTTTATCTGCTATAGCCTGCAG
81	YKF-16_463418_16	Deeplab-GH10-81-F	ATGAGAAAGAACAACAAAGGTATCTGG
	YKF-16_463418_16	Deeplab-GH10-81-R	CTACTTGGAGTAGAGCTGCATG
82	YKF-16_1785338_2	Deeplab-GH10-82-F	ATGGATTTCCTTAAAACTGATGATTCTC
	YKF-16_1785338_2	Deeplab-GH10-82-R	TTACCCTAGATAAGTAATTTCGAATTTA GTTG
83	YKM-01_533181_53	Deeplab-GH10-83-F	ATGAATAGAATCTTTAGAAACAGTC
	YKM-01_533181_53	Deeplab-GH10-83-R	TTATTGTCCACTAAGAGCATCG
84	YKM-01_790390_5	Deeplab-GH10-84-F	ATGGATACGACCAACAAAAAAG
	YKM-01_790390_5	Deeplab-GH10-84-R	CTATAATGATATCATCGAGAGATTG
85	YKM-02_303379_6	Deeplab-GH10-85-F	ATGAAGACGACATTTGCGACAACTG
	YKM-02_303379_6	Deeplab-GH10-85-R	TCATTCCACCTCGACGAACTTG
86	YKM-02_482037_28	Deeplab-GH10-86-F	ATGGATGAGCTGAAGCATCGC
	YKM-02_482037_28	Deeplab-GH10-86-R	TTATGCCAGCGTCAGCGTCTG
87	YKM-03_1260944_2	Deeplab-GH10-87-F	ATGAAACATTATTCAAAGATTGCCCTCG
	YKM-03_1260944_2	Deeplab-GH10-87-R	TCACCCGTCATACGTCTTGCTG
88	YKM-03_1454588_2	Deeplab-GH10-88-F	ATGCCGGGACTCAAGGATGTG
	YKM-03_1454588_2	Deeplab-GH10-88-R	TTATTTGGGTATCGCGGCGACG
89	YKM-11_421200_16	Deeplab-GH10-89-F	ATGAAAAGATTCGTGTTATTGACCC
	YKM-11_421200_16	Deeplab-GH10-89-R	TCATAAGTCAACAATTGCAGTATGTTC
90	YKM-11_432829_10	Deeplab-GH10-90-F	ATGAAAGTTTCACGTATATTGGGC
	YKM-11_432829_10	Deeplab-GH10-90-R	CTATTCTGGAATTGCCAATAAGCGG
91	YKM-12_294961_3	Deeplab-GH10-91-F	ATGAAAAAAACAGCATTGATTGCCGC
	YKM-12_294961_3	Deeplab-GH10-91-R	CTATTCCAGCTCCGCGAAGG
92	YKM-12_1442233_1	Deeplab-GH10-92-F	ATGAATAGAACAGGATTACTCCTAC
	YKM-12_1442233_1	Deeplab-GH10-92-R	TTACCCGCATAATTCAGTGACAG
93	YKM-13_1013813_2	Deeplab-GH10-93-F	ATGAATAAGAAAGCACTTATTGCAATC
	YKM-13_1013813_2	Deeplab-GH10-93-R	TTATTGTTTTTGAATCTTCTTTCCGTTG

94	YKM-13_1127811_2	Deeplab-GH10-94-F	ATGGTTAAACAGTTTCTTATATTATTTC
	YKM-13_1127811_2	Deeplab-GH10-94-R	TTATCCTAAATTGGCTTTAGCAGC
95	CYM-02_302522_2_F	Deeplab-GH10-95-F	ATGCCTTTTTTATATAAAGTACGTATC
	CYM-02_302522_2_R	Deeplab-GH10-95-R	TTAGAATGATTTCACCACCTTCTTG
96	CYM-02_682691_6_F	Deeplab-GH10-96-F	ATGAAGACGACATTGGCGACAAC
	CYM-02_682691_6_R	Deeplab-GH10-96-R	TCACTCGACCTCGACGAACTTG
97	CYF-14_533661_2	Deeplab-GH10-97-F	ATGGAGCAGCTCAAACACCG
	CYF-14_533661_2	Deeplab-GH10-97-R	TCAGATGAGGGTCAAAGACGC
98	CYF-16_489294_1	Deeplab-GH10-98-F	ATGAAAGTCAGAAATTTATATCTTTTATTC
	CYF-16_489294_1	Deeplab-GH10-98-R	TTACTCTAGATATTTAATTTCGAATTTGG
99	CYM-02_483816_2	Deeplab-GH10-99-F	ATGAAAAAACTTTCAACAATCGCTC
	CYM-02_483816_2	Deeplab-GH10-99-R	TTACTTCTTGAAATCGCGTACTGTG
100	CYM-12_1153444_7	Deeplab-GH10-100-F	ATGAAAAAGATATTTATAATCTCTGTAG
	CYM-12_1153444_7	Deeplab-GH10-100-R	CTACTGTCCGCTAAGCGCC
101	CYM-13_416286_12	Deeplab-GH10-101-F	ATGAAGGCAGGGTGTATATCGG
	CYM-13_416286_12	Deeplab-GH10-101-R	TCATATGGCGATCGTTGTCCC
102	HCM-02_284089_2	Deeplab-GH10-102-F	ATGGATATAACACACAGACTGGCC
	HCM-02_284089_2	Deeplab-GH10-102-R	TTAGTTTTCCAATAACTTAATGCTCTC
103	HCM-11_355688_2	Deeplab-GH10-103-F	ATGGATAAATTTAATCACAGAAAAGC
	HCM-11_355688_2	Deeplab-GH10-103-R	CTACAAGATGATCTCTTTGACGG
104	HCM-11_725930_6	Deeplab-GH10-104-F	ATGAACACGCGAAGGGAGTTTC
	HCM-11_725930_6	Deeplab-GH10-104-R	CTAAACCTTGATCTCGATCCTGTC
105	LCF-04_16054_23	Deeplab-GH10-105-F	ATGATGAAAAAAGCTTTGCTCATGC
	LCF-04_16054_23	Deeplab-GH10-105-R	TCACCGCTTCGGTTGCTTG
106	LCF-04_733572_3	Deeplab-GH10-106-F	ATGCTTGAGCTAAAGCGAAAAATTATG
	LCF-04_733572_3	Deeplab-GH10-106-R	TTACTTTGCGGTGAGGCCTTC
107	LCF-14_704985_1	Deeplab-GH10-107-F	ATGTCCTACGAGCATCGCAAAG
	LCF-14_704985_1	Deeplab-GH10-107-R	TTATTCCAGCGTGATTTCGATGTC
108	LCF-15_80711_2	Deeplab-GH10-108-F	ATGAATAAGAGTTTTAGAAACAGTCTC
	LCF-15_80711_2	Deeplab-GH10-108-R	TTATTTTCCACCGAATGCATCGC
109	LCF-15_328285_1	Deeplab-GH10-109-F	ATGTCCTACGAGCATCGCAAAG
	LCF-15_328285_1	Deeplab-GH10-109-R	TTACAGCGTCACATCCAGCG
110	LCM-11_465898_2	Deeplab-GH10-110-F	ATGAAAAATCGTTCATTTCTTCCCG
	LCM-11_465898_2	Deeplab-GH10-110-R	CTAGTGAACGACCGTCTCAAG
111	LCM-12_657468_7	Deeplab-GH10-111-F	ATGGACAAATATATGCACCGGAAAG
	LCM-12_657468_7	Deeplab-GH10-111-R	TTAACGGGCAAGAGTGACTGTTC
112	YKF-14_667271_3	Deeplab-GH10-112-F	ATGTCCTACGAGCATCGCAAAG

YKF-14_667271_3	Deeplab-GH10-112-R	TTACAGCGTGACCTCCAGC

Note: Results not verified by PCR are marked in red.
