# Supplementary material for: Dietary Conversion from All-Concentrate to All-Roughage Alters Rumen Bacterial Community Composition and Function in Yak, Cattle-Yak, Tibetan Yellow Cattle and Yellow Cattle
Source: Animals (Basel). 2024 Oct 11;14(20):2933. doi: 10.3390/ani14202933 (PMC11503692; doi:10.3390/ani14202933)
Supplement: Supplementary file 1 [file animals-14-02933-s001.zip › Table S5-Rumen microbial abundance table at genus level.pdf]

Table S5 Rumen microbial abundance table at genus level

	Phylum level	Concentrate	Roughage	SEM	P-value
YK	*Butyrivibrio*	0.0016[b]	0.0038[a]	0.0005	0.010
	Eubacterium	0.0021	0.0022	0.0001	0.895
	Fibrobacter	0.0124	0.0085	0.0028	0.520
	Methanobrevibacter	0.0172	0.0212	0.0017	0.235
	Mogibacterium	0.0028	0.0025	0.0003	0.721
	Prevotella	0.1177	0.1123	0.0068	0.713
	Ruminobacter	0.0048	0.0005	0.0014	0.114
	Ruminococcus	0.0431[a]	0.0096[b]	0.0078	0.023
	Sarcina	0.0051	0.0036	0.0005	0.113
	Selenomonas	0.0021	0.0010	0.0003	0.134
	Succinivibrio	0.0003	0.0005	0.0001	0.508
	Treponema	0.0048	0.0043	0.0008	0.774
CY	*Butyrivibrio*	0.0018	0.0030	0.0005	0.292
	Eubacterium	0.0020	0.0024	0.0003	0.412
	Fibrobacter	0.0025[b]	0.0126[a]	0.0021	0.008
	Methanobrevibacter	0.0130	0.0178	0.0024	0.330
	Mogibacterium	0.0030	0.0033	0.0006	0.823
	Prevotella	0.1408	0.1091	0.0173	0.384
	Ruminobacter	0.0047[a]	0.0008[b]	0.0010	0.039
	Ruminococcus	0.0493[a]	0.0078[b]	0.0084	0.006
	Sarcina	0.0067	0.0054	0.0011	0.591
	Selenomonas	0.0022	0.0011	0.0003	0.084
	Succinivibrio	0.0006	0.0020	0.0006	0.226
	Treponema	0.0033	0.0054	0.0011	0.368
HC	*Butyrivibrio*	0.0015	0.0028	0.0006	0.274
	Eubacterium	0.0021	0.0026	0.0002	0.273
	Fibrobacter	0.0206	0.0279	0.0065	0.594
	Methanobrevibacter	0.0080	0.0103	0.0012	0.382
	Mogibacterium	0.0023	0.0016	0.0002	0.130
	Prevotella	0.2035[b]	0.1363[a]	0.0109	0.000
	Ruminobacter	0.0059	0.0009	0.0013	0.062
	Ruminococcus	0.0253	0.0153	0.0027	0.057
	Sarcina	0.0041	0.0030	0.0005	0.350
	Selenomonas	0.0033	0.0012	0.0006	0.068
	Succinivibrio	0.0010	0.0096	0.0043	0.340
	Treponema	0.0051	0.0031	0.0007	0.168
LC	*Butyrivibrio*	0.0018	0.0039	0.0006	0.072
	Eubacterium	0.0020[b]	0.0036[a]	0.0004	0.034
	Fibrobacter	0.0055[b]	0.0123[a]	0.0017	0.040
	Methanobrevibacter	0.0166	0.0206	0.0019	0.306
	Mogibacterium	0.0027	0.0027	0.0003	0.960
	Prevotella	0.1784	0.1291	0.0159	0.125

Ruminobacter	0.0090	0.0003	0.0031	0.170
Ruminococcus	0.0285[a]	0.0105[b]	0.0042	0.024
Sarcina	0.0042	0.0031	0.0005	0.352
Selenomonas	0.0026[a]	0.0013[b]	0.0003	0.031
Succinivibrio	0.0004	0.0008	0.0002	0.277
Treponema	0.0036	0.0062	0.0020	0.554

Note: Peer data with no letter shoulder indicates no significant difference ($P > 0.05$), while peer data with lower-case letter shoulder indicates significant difference ($P < 0.05$).
