# Supplementary material for: Dietary Conversion from All-Concentrate to All-Roughage Alters Rumen Bacterial Community Composition and Function in Yak, Cattle-Yak, Tibetan Yellow Cattle and Yellow Cattle
Source: Animals (Basel). 2024 Oct 11;14(20):2933. doi: 10.3390/ani14202933 (PMC11503692; doi:10.3390/ani14202933)
Supplement: Supplementary file 1 [file animals-14-02933-s001.zip › Table S6-The number of functional genes encoding different CAZymes in the concentrate and roughage groups.pdf]

Table S6. The number of functional genes encoding different CAZymes in the concentrate and roughage groups

**Groups**	C	R
YK	252	258
CY	247	258
HC	244	257
LC	245	255
