# Supplementary figures and images for: Dietary Conversion from All-Concentrate to All-Roughage Alters Rumen Bacterial Community Composition and Function in Yak, Cattle-Yak, Tibetan Yellow Cattle and Yellow Cattle
Source: Animals (Basel). 2024 Oct 11;14(20):2933. doi: 10.3390/ani14202933 (PMC11503692; doi:10.3390/ani14202933)

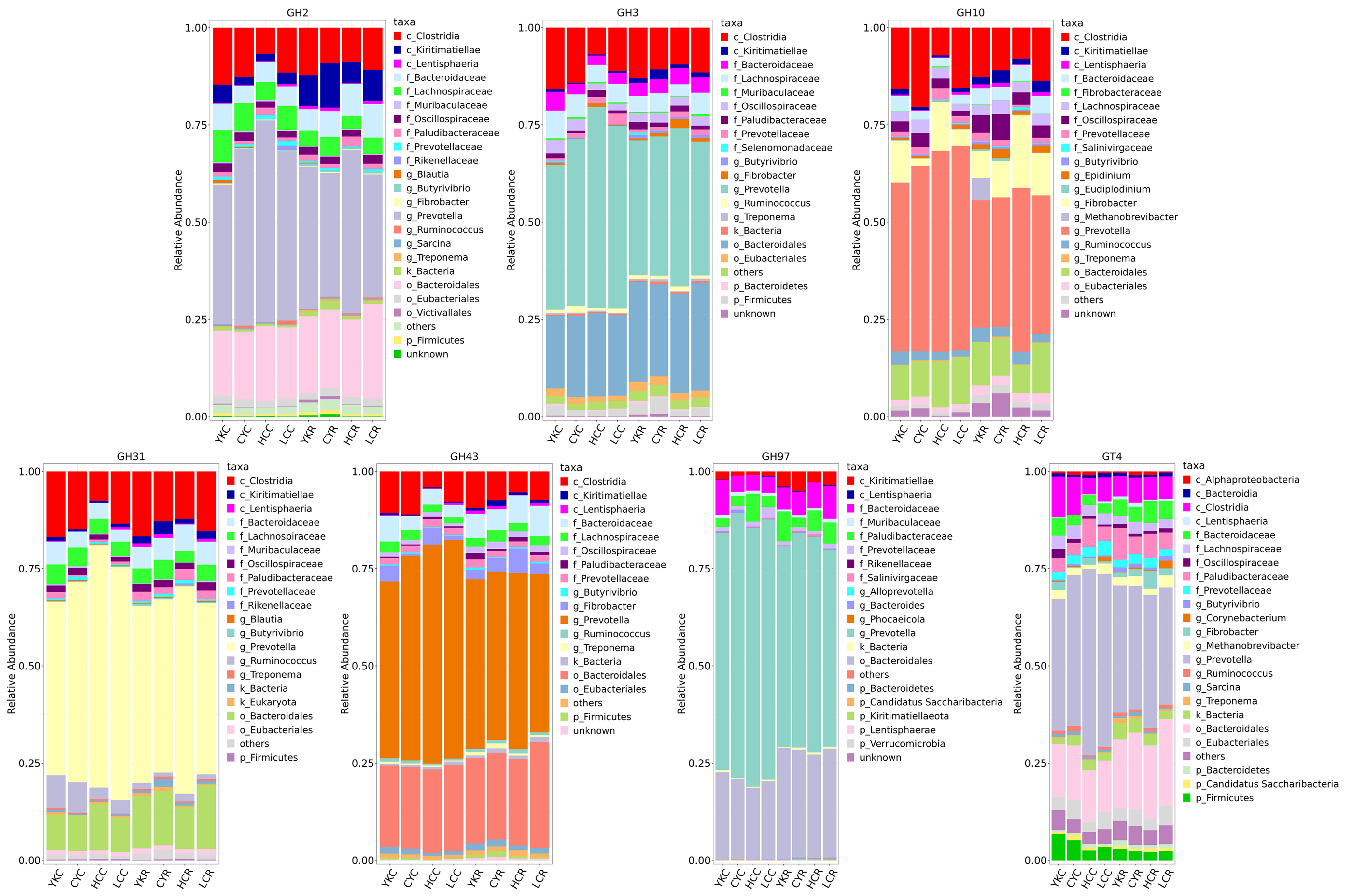

Supplement: Supplementary file 1 [file animals-14-02933-s001.zip › Figure S1-Relative contribution of different taxa to identified rumen-enriched functional attributes of CAZymes encoded genes at the family level in different samples.jpg]

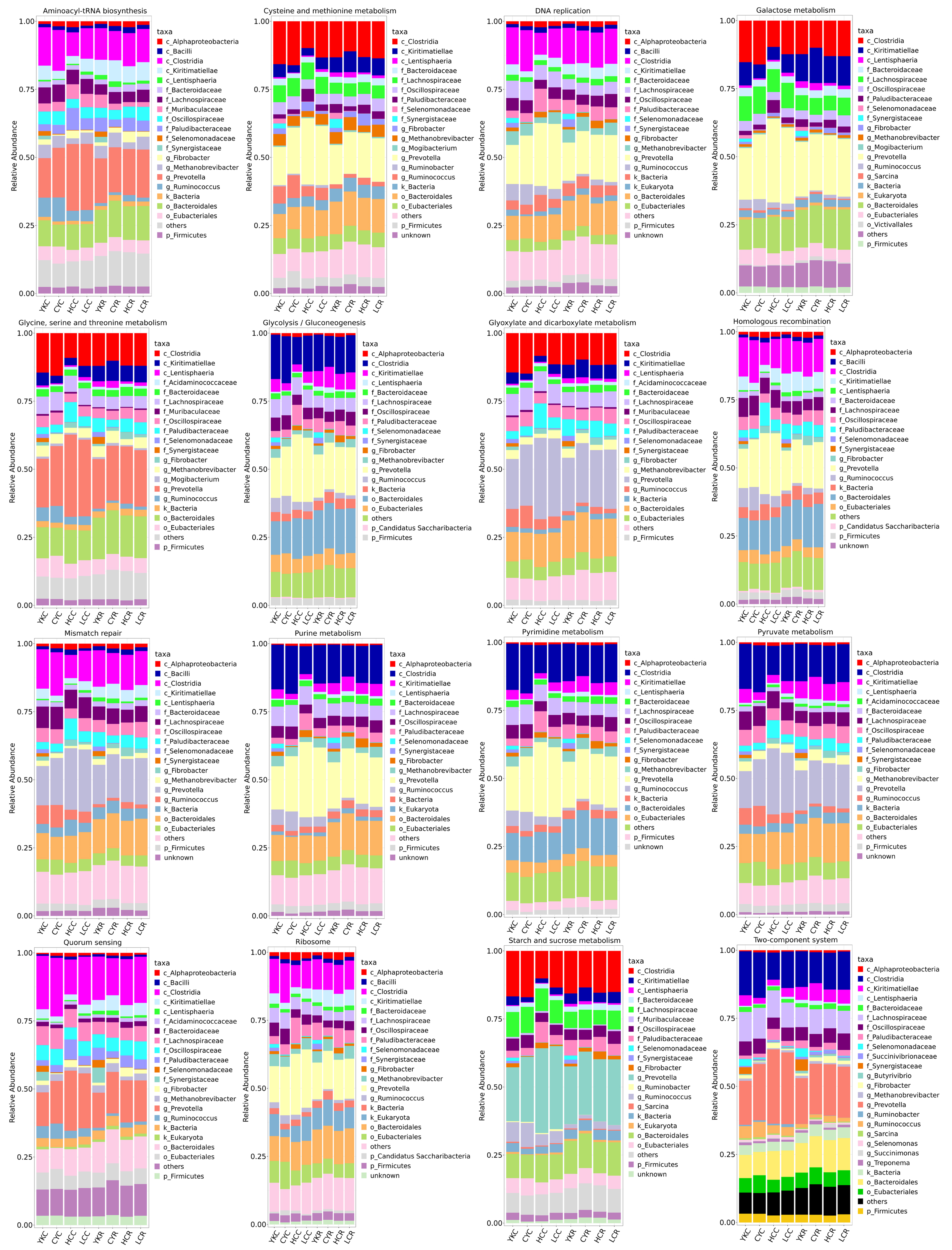

Supplement: Supplementary file 1 [file animals-14-02933-s001.zip › Figure S2-Relative contribution of different taxa to identified rumen-enriched functional attributes of KEGG pathway level 3 in different samples.jpg]
